# From Green to Ripe: Untargeted UHPLC–Orbitrap–MS Lipidomic Profiling of Cacao Seed and Pod Husk during Maturation

**DOI:** 10.1021/acs.jafc.6c08470

**Published:** 2026-08-29

**Authors:** João V. M. de Almeida, Daniel S. Trancoso, Eclair V. Filho, Alan R. Pereira, Anna C. F. Braz, Nathália dos S. Conceição, Julie M. M. Figueiredo, Marcio V. Rodrigues, Nayara A. dos Santos, Ricardo M. Kuster, Wanderson Romão

**Affiliations:** † Federal University of Espírito Santo/UFES, Department of Chemistry, Campus Goiabeiras, Avenida Fernando Ferrari, 514, Vitoria, Espírito Santo 29075-910, Brazil; ‡ Federal University of Espírito Santo/UFES, Department of Pharmaceutical Sciences, Campus Maruipe, Avenida Marechal Campos, 1468, Vitoria, Espírito Santo 29043-213, Brazil; § Federal Institute of Espírito Santo/IFES, Campus Vila Velha, Av. Ministro Salgado Filho, Vila Velha, Espírito Santo 29106-010, Brazil; ∥ Federal Institute of Espírito Santo/IFES, Campus Linhares, Av. Filogônio Peixoto, Linhares, Espírito Santo 29901-291, Brazil; ⊥ Federal Institute of Espírito Santo/IFES, Campus Vitória, Av. Vitória, Vitória, Espírito Santo 29040-780, Brazil

**Keywords:** *Theobroma cacao* L., LC-MS, lipid metabolism, plant biochemistry, ripening.

## Abstract

Cacao fruit maturation directly influences the chemical composition of tissues that are central to chocolate production and byproduct valorization, yet its lipid remodeling remains unexplored. Here, an untargeted UHPLC–Orbitrap–MS lipidomics workflow was used to map lipid profile changes in cacao seeds and cacao pod husk (CPH) across three maturation stages (green, intermediate, and ripe). In seeds, 290 lipid species were annotated, with most lipid classes showing lower relative abundance at the mature stage. Major cocoa butter triacylglycerols exhibited selective accumulation, revealing that maturation involves targeted lipid reorganization rather than a generalized increase in storage lipids. In CPH, 224 lipid species were annotated, with limited variation at the class level but pronounced species-specific changes; steryl esters, diacylglycerols, hydroxy fatty acids, and acylated sterol glycosides increased during ripening. These trends indicate distinct metabolic roles for storage and protective tissues during cacao development.

## Graphical abstract

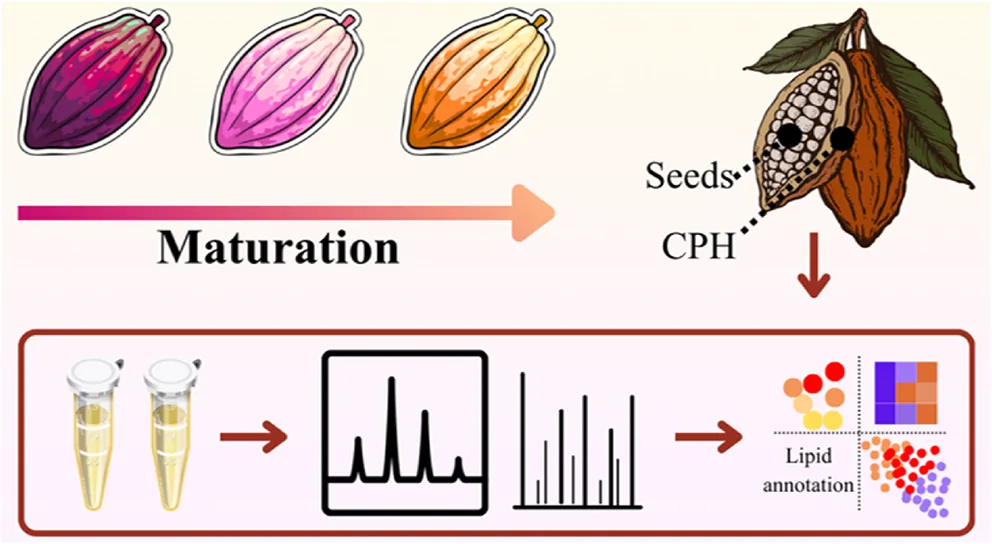

## 1 Introduction

From the tropical rainforests of the Americas, the cacao plant (*Theobroma cacao* L.), belonging to the *Malvaceae* family, is a neotropical evergreen tree, with a history of 5,300 cal. yr BP with humans.^1–4^ The fruit comprises cacao pod husk (CPH), seeds, and pulp, with the economic importance of cacao being predominantly linked to the seeds, followed by the pulp, which makes cacao one of the world’s top ten agricultural commodities.^2,5,6^

Cacao seeds are a complex matrix containing various components, many of which have been associated with biological activities.^7,8^ They undergo extensive processing, from fermentation to roasting and grinding.^7^ Cocoa (dried, fermented beans) contains lipids, carbohydrates, bioactive compounds such as polyphenols and methylxanthines, as well as proteins, fiber, and minerals, whereas the cocoa shell can contain similar classes in different relative abundances.^7,9,10^

Lipids are the most abundant seed components, representing over half of the seed dry weight and varying with origin, genotype, and climate.^11,12^ In cocoa butter (CB), around 97% of these lipids are glycerolipids (GL), composed mainly of triacylglycerols (TG), whereas the remaining 3% consists of glycolipids and unsaponifiable matter.^12,13^ Cacao fat contributes to CB organoleptic, rheological, and crystallization properties, while also being essential for membrane structure, energy storage, and metabolic signaling in plants; however, their role in flavor and bioactive properties is not fully understood.^12,14,15^

In this context, understanding the lipid composition of cacao seeds is relevant to both agriculture and the food industry, as lipids play important roles in plant growth and responses to environmental conditions, while also contributing to authenticity assessment, traceability, and food quality.^16–18^ They may also exhibit biological activities; thus, identifying new species and elucidating their occurrence and roles in plant tissues and food matrices are of considerable interest.^12^ Finally, advances in understanding cacao plant biology can facilitate innovations in plant-based products.^18^

In this setting, mass spectrometry (MS) is the most used instrument for lipid analysis, with many studies focusing on liquid chromatography–mass spectrometry (LC-MS).^18,19^ Its use for cacao lipidomics mainly focuses on seeds, lipid changes during postharvest processing, and characterization of TGs.^12,20–24^ In this context, Herrera-Rocha et al. (2022) conducted an untargeted lipidomics study during fermentation, evaluating time zero, and found no significant changes.^12^ Despite advances, studies focusing on lipids in fresh fruit tissues, particularly CPH, remain scarce.

In addition to the limited lipidomics studies on fresh fruit, changes in metabolite composition during maturation are underexplored, showing a gap in the knowledge of cacao fruit biochemistry, since characterizing changes in key metabolites during ripening is essential for elucidating tissue-specific metabolic regulation and its potential implications for fruit quality and biological function.^25,26^ Furthermore, given the amount of CPH generated as waste, identifying compounds of potential industrial interest contributes to its valorization and the development of new applications.^10^

Thus, the goal of this study was to perform an untargeted lipidomics workflow to investigate the lipid composition of cacao seeds with pulp and CPH during maturation, using ultrahigh-performance liquid chromatography–mass spectrometry (UHPLC-MS) to elucidate biochemical alterations associated with ripening.

## 2 Materials and Methods

### 2.1 Chemicals and Materials

LC-MS acetonitrile (ACN), methanol (MeOH), and HPLC-grade isopropanol (IPA) came from Merck (Darmstadt, HE, Germany). Chloroform was obtained from Sigma-Aldrich (St. Louis, MO, USA). Formic acid (98%) was supplied by NEON (Suzano, SP, Brazil). Ammonium formate (>99.5%) was purchased from Fisher Chemical (Pittsburgh, PA, USA). Water was purified using a Milli-Q purification system (18.2 MΩ·m) (Milford, MA, USA). Syringe filters with a PTFE pore size of 0.45 μm and 13 mm diameter were from Filtrilo (Colombo, PR, Brazil).

### 2.2 Sample Preparation and Controls

Forty-five fruits of the PS1319 clone were harvested from a full-sun experimental plantation at Fazenda São Luiz (Linhares, ES, Brazil; 19.45676° S, 39.87775° W), where clonal plants are cultivated in close spacing without shade. The fruits were collected from >10 cacao trees and separated into three maturation stages (n = 15 per stage). The stages were initially defined at harvest based on the external husk color, the inner husk color after scraping the outer surface, and were further supported after fruit opening by the qualitative internal consistency observed within each group.

Green fruits (E1) had an intense external purple coloration with a green inner husk after scraping, with the pulp/mucilage visually occupying a larger internal space relative to the seed mass and with no clear separation between the seed–pulp mass and the inner pod wall. Intermediate fruits (E2) showed variable external coloration, ranging from purple to yellow, with a few fruits showing yellow-orange tones, a characteristic yellowish-green inner husk after scraping, and transitional internal characteristics, including a more balanced occupation between pulp/mucilage and seed mass and progressive separation from the inner pod wall. Ripe fruits (E3) had predominantly yellow-orange external coloration with red tones, with well-formed seeds occupying a larger proportion of the internal fruit space, softer pulp/mucilage, and clearer separation between the seed–pulp mass and the husk.

CPHs were separated from cacao seeds with pulp (analyzed together and hereafter referred to as “cacao seeds”). Three to six cacao seeds (from the ends and middle regions of each fruit) and three to five small portions of the CPH, picked from random fruit location, were frozen at −80 °C for 1 h and freeze-dried for 24 h at −44 °C using an LS3000 Terroni (São Carlos, SP, Brazil). Seeds were ground using a mini food processor and a grain grinder, whereas CPH was ground only with the latter.

For each sample, 15 mg of the material was weighed, and 600 μL of MeOH:chloroform (1:2 v/v) was added to a 1.5 mL Eppendorf tube (Hamburg, Germany). The samples were vortexed for 30 s and sonicated for 20 min. After extraction, the mixtures were centrifuged for 6 min at 21,300 × g and 23 °C in a 5425 R centrifuge (Eppendorf, Hamburg, Germany). The supernatants were then filtered through a 0.45 μm membrane filter and dried in a vacuum concentrator (Concentrator Plus, Eppendorf, Hamburg, Germany) at 45 °C for 1 h (V-AQ mode). The dried extracts were reconstituted in 1000 μL of MeOH:isopropanol (IPA) (1:3, v/v), and 550 μL were transferred to 1 mL vials and injected into a UHPLC-MS system (ESI (+): 1 μL; ESI (−): 4 μL for seeds; ESI (+): 3 μL; ESI (−): 5 μL for CPH). A Quality control (QC) containing 13 μL of each sample, and a solvent blank (reagent blank) were analyzed in quintuplicate. Three preparation blanks (method blanks) were generated along with the samples for five blank analyses in each sequence. A quality control (QC) containing 13 μL of each sample, and a solvent blank (reagent blank) were analyzed in quintuplicate. Three preparation blanks (method blanks) were generated along with the samples for a total of five blank analyses in each sequence.

### 2.3 Instrumentation

A Vanquish UHPLC system (Thermo Fisher Scientific, United States) equipped with a Luna Omega C18 100 Å column (2.1 × 150 mm, 1.6 μm) was used, with phase A (0.1% formic acid and 10 mmol L^–1^ ammonium formate in H_2_O:ACN (40:60, v/v), and phase B (0.1% formic acid and 10 mmol L^–1^ ammonium formate in IPA:ACN:H_2_O (90:9.5:0.5, v/v), and a flow rate of 0.3 mL min^–1^. The elution gradient was: 5% of B from 0.0 to 1.0 min (curve-5), increasing to 45% in 1.0 min (curve-6), then rising from 45% to 85% over 0.5 min (curve-5), and from 85% to 100% over 4.0 min (curve-9), with 100% maintained for 5.1 min (curve-5). Finally, the conditions returned to the initial parameters over 0.4 min and were held for an additional 2.0 min. The column temperature was maintained at 45 °C, and the sampler module was at 25 °C.

For data acquisition, an Orbitrap Exploris 120 (Thermo Fisher Scientific, United States) was used with an electrospray ionization (ESI) source operating in negative and positive modes separately. A full-scan mode with an *m*/*z* range of 100–1500 at a resolution of 120,000, using internal calibration with Easy-IC (RunStart) was implemented. Data-dependent acquisition was performed using HCD with a normalized collision energy (NCE) of 50%, resolution of 15,000, and with a dynamic exclusion method (exclude after two times if it occurs within 1 s; duration of 10 s). For TGs, a QC sample was analyzed in ESI (+) in the same method, except for a normalized collision energy (NCE) of 30%. The ESI parameters in Table S1.

The injection sequence was organized as follows: solvent blank, QC, preparation blank, nine samples, and the pattern was repeated throughout the sequence. Samples were ordered by maturation, starting with E1. Volume injected: ESI (+): 1 μL; ESI (−): 4 μL for seeds; ESI (+): 3 μL; ESI (−): 5 μL for CPH.

### 2.4 Data Processing and Lipid Identification

Data were processed in MS-DIAL 5.5, with an MS^1^ mass tolerance of 0.01 and 0.05 Da for MS,^2^ linear weighted moving average smoothing method, no MS/MS cutoffs, and with gap filling by compulsion and weight (0.5) for Retention time (Rt) and MS^1^ factors. The remaining parameters are shown in Table S2.^27^ For annotation, the internal MS-DIAL library was used along with “MoNA-export-LipidBlast_2022”.^28^

Compound Discoverer v3.5 (Thermo Fisher Scientific, Waltham, MA, USA) was used solely for annotation using the template “LC/Lipidomics/Untargeted Lipidomics using LipidSearch (MKI)”. The parameters used were based on MS-DIAL values, while nonspecified parameters were kept at their default settings.

Additionally, the Global Natural Products Social Molecular Networking 2 (GNPS) platform was utilized with exported data from MS-DIAL for annotation only. Connections between nodes were established using an MS^2^ cosine similarity cutoff of 0.7, with clusters limited to 50 nodes.^29^ To match with GNPS libraries, a cosine threshold of 0.8 was used. Mass tolerances were set at 0.01 and 0.05 Da.

### 2.5 Statistical Analysis

Statistical analysis was carried out using MetaboAnalyst 6.0 with exported data from MS-DIAL, excluding features without MS.^2,30^ After the QC Relative Standard Deviation (RSD) calculation, only samples from the maturation stages were evaluated. For each sample group (seeds and CPH) and ionization mode (+ and −), two data sets were generated: (1) all features with MS/MS information and (2) the summed raw intensities (peak areas) of annotated compounds within each lipid class. The normalization parameters are provided in Table S2.

Principal Component Analysis (PCA) and Partial Least Squares Discriminant Analysis (PLS-DA) were applied to data sets (1). A one-way ANOVA (*p*-value (FDR) cutoff of 0.05) was performed for data sets (1) to evaluate the statistical significance of annotated features, and data sets (2) to evaluate all lipid classes. Boxplots were generated for the main features of data set (1) and for all classes with *p*-values < 0.05 (2). Heatmaps (normalized data; auto scale features; Euclidean; ward clustering; group averages) were generated for lipid classes showing statistical significance in data sets (2) for the whole group and each class to see changes across the groups.

Carryover was assessed using feature-wise RSD analysis and Spearman correlation between feature intensities and injection order, based on MS-DIAL peak-area exports of solvent blanks (1–5) without applying the blank-filtering parameter, in Microsoft Excel.

## 3 Results and Discussion

### 3.1 Annotation and Method Evaluation

In seeds, 290 lipids were annotated, 114 only in ESI (+), 159 only in ESI (−), and 17 in both, divided into 22 classes (Table S4, Figure 1). In CPH, 224 lipids were annotated, 86 only in ESI (+), 121 only in ESI (−), and 17 in both, divided into 23 classes (Table S5, Figure 1), with 59.56% of the same lipids as the seeds. Annotations were supported by MS/MS and correspond to level 2 of metabolite identification.^31^ Tables S4 and S5 include the *p*-values from one-way ANOVA, with a significance threshold of 0.05. These *p*-values refer to the global ANOVA comparison among maturation stages, indicating whether each annotated lipid feature showed significant changes across groups.

**Figure 1:**
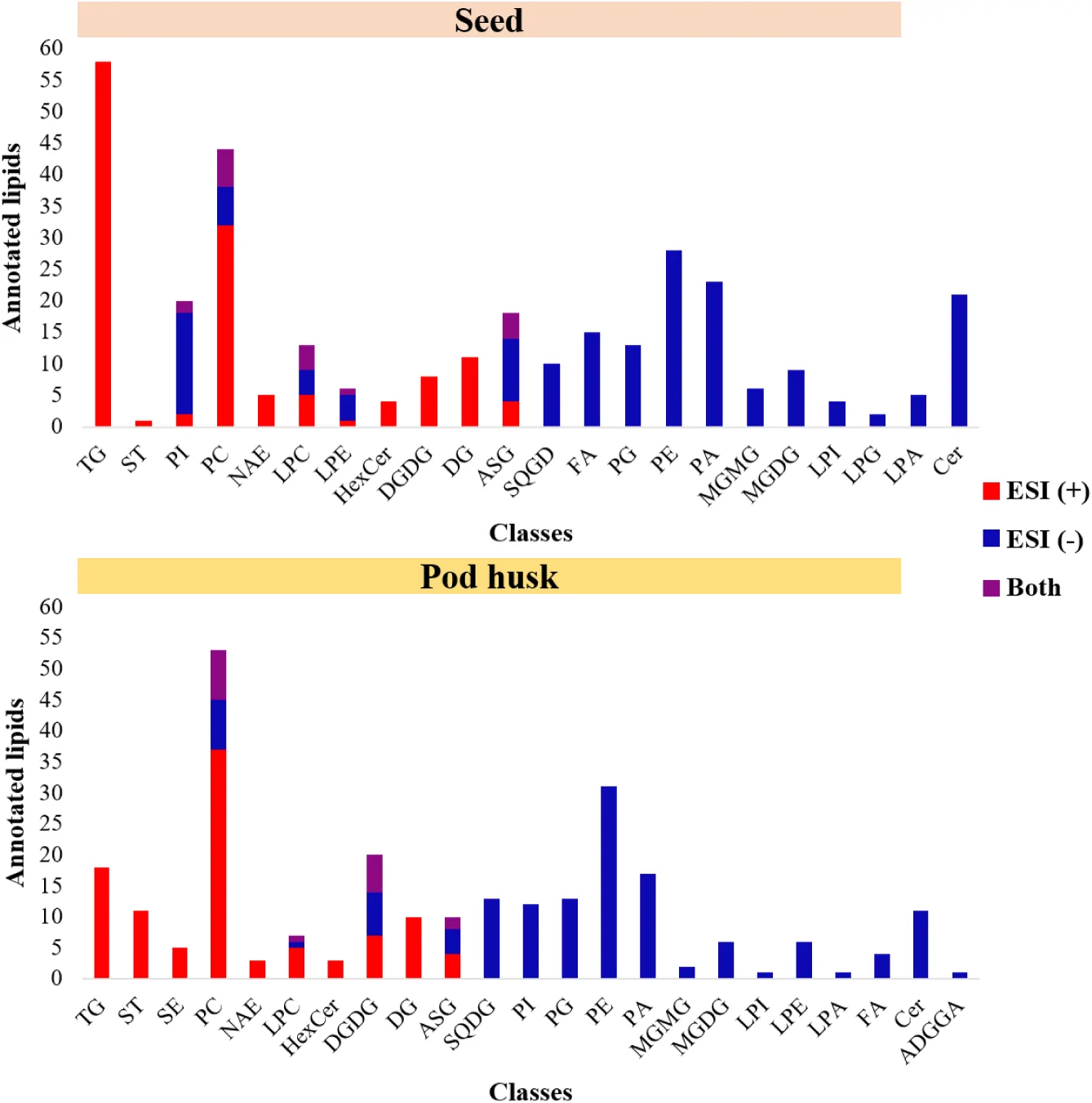
Number of annotated lipids in each class for cacao seeds and CPH. ST – Sterols; PI – Phosphatidylinositol; PC – Phosphatidylcholine; NAE – N-Acylethanolamine; LPC – Lysophosphatidylcholine; LPE – Lysophosphatidylethanolamine; HexCer – Hexosylceramide; DGDG – Digalactosyldiacylglycerol; DG O- – Ether-linked Diacylglycerol; DG – Diacylglycerol; ASG – Acylated Sterol Glycoside; SQDG – Sulfoquinovosyldiacylglycerol; FA – Fatty Acid; PG – Phosphatidylglycerol; PE – Phosphatidylethanolamine; PA – Phosphatidic Acid; MGMG – Monogalactosylmonoacylglycerol; MGDG – Monogalactosyldiacylglycerol; LPI – Lysophosphatidylinositol; LPG – Lysophosphatidylglycerol; LPA – Lysophosphatidic Acid; Cer – Ceramide; ADGGA – Acyl-Diacylglyceryl Glucuronide; SE – Steryl Ester.

The first and last QC chromatograms (Figure 2) displayed lipid class distribution in cacao seeds and CPH profiles, with peaks showing consistent retention times. Additionally, MetaboAnalyst RSD comparison (Figure S1) demonstrated good analytical stability, since QC variation was significantly lower than sample variation. This suggests that observed differences were mainly due to sample variation rather than instrumental variability. Before filtering, solvent blank analysis revealed that 18%, 31%, 27%, and 31% of background features in seed ESI (+), seed ESI (−), CPH ESI (+), and CPH ESI (−), respectively, exhibited monotonic trends (|ρ| ≥ 0.7). Since solvent blank injections did not show a corresponding increase in signal, these findings indicate no statistically relevant carryover.

**Figure 2:**
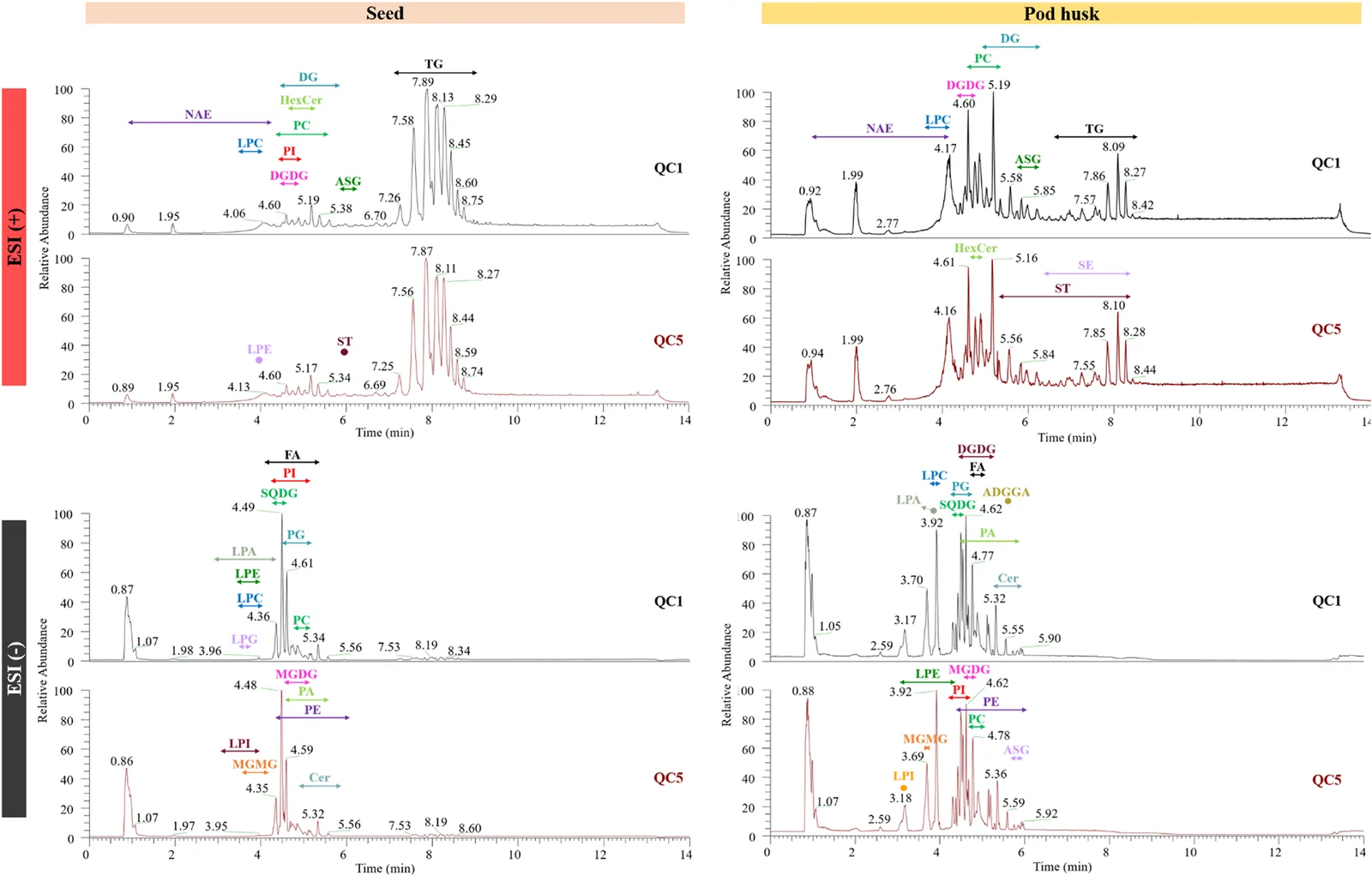
First and last QCs chromatograms of the seeds and CPH analysis.

### 3.2 General Chemometric and Statistical Analysis

PCA and PLS-DA were performed on cacao seeds and CPH samples across maturation stages using all MS/MS features (Figure 3). PCA of the seeds did not show a clear clustering pattern among maturation stages, indicating that the global metabolomic profiles remained broadly similar throughout seed development. However, a slight tendency for E1 to separate from the later stages was observed, suggesting that early maturation may involve subtle metabolic differences. The substantial within-group variation, particularly in the earlier stages, likely contributed to the overlap among groups, and the reduced dispersion in E3 suggests a more homogeneous metabolomic profile at the final stage of maturation. Furthermore, PLS-DA successfully separated E1 from E2 and E3, with greater similarity between the last two stages. For CPH, the PCA model showed better separation between groups than seeds, with ESI (+) clustering E1 and E2, and ESI (−) clustering E1 similarly. Finally, PLS-DA in ESI (+) showed the best visual separation among groups, whereas in ESI (−), E1 remained separated, E2 showed greater variation, and E3 appeared closer to E2. In some models, component 3 was used instead of component 2 because it captured more relevant variance for group discrimination. In summary, E1 was consistently distinguished from E3 across all models, supporting metabolic changes between early and late maturation stages.

**Figure 3:**
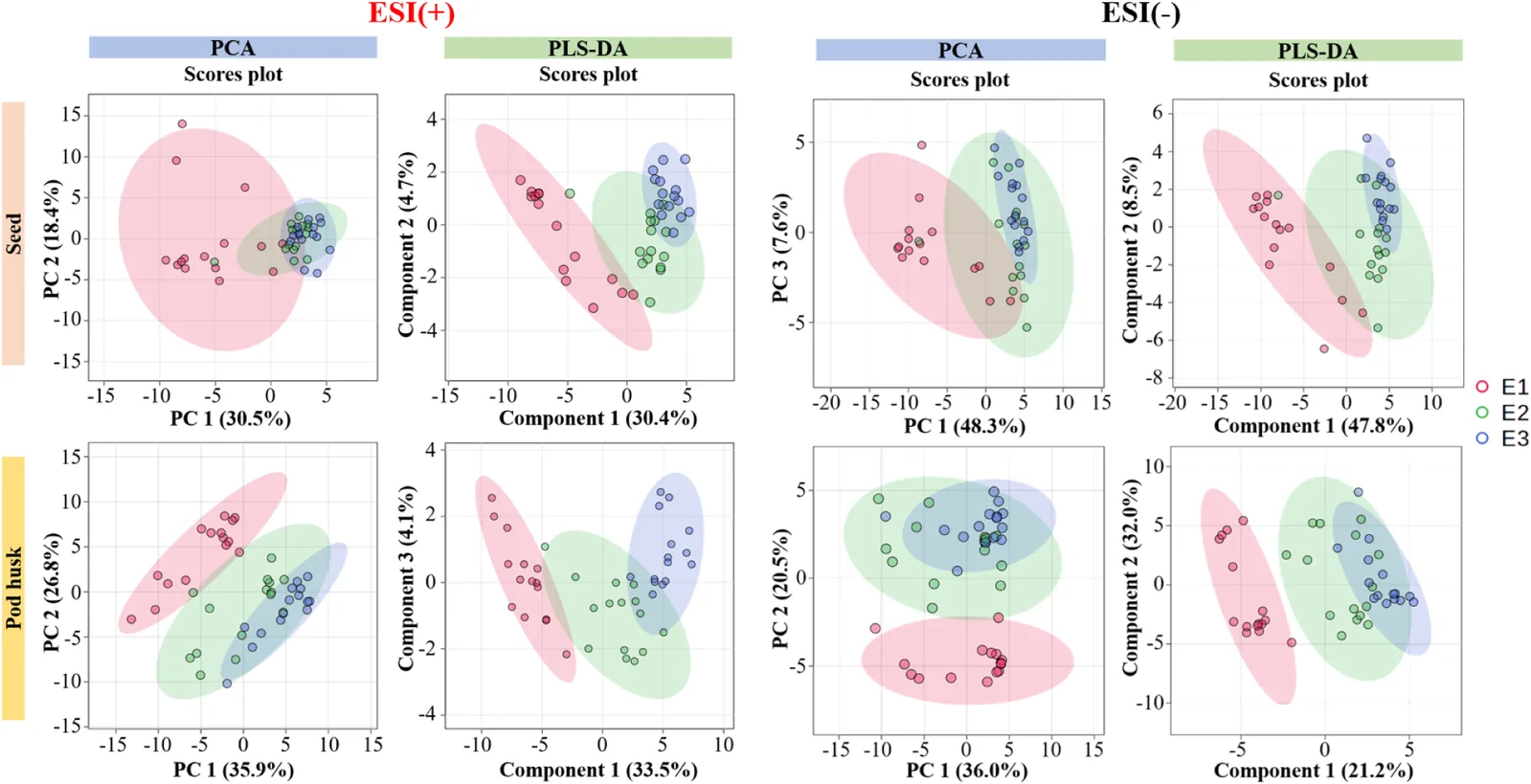
PCA (*p*-value < 0.05) and PLS-DA score plots of lipid profiles from cacao seeds and CPH at different maturation stages in ESI (+) and ESI (−) modes. PLS-DA cross validations (2 comps): seeds (ESI (+) – Q^2^ (0.70672) R^2^ – (0.88488); ESI (−) – Q^2^ (0.78475) R^2^ – (0.84897)) CPH (ESI (+) – Q^2^ (0.83062) R^2^ – (0.87049); ESI (−) – Q^2^ (0.78522) R^2^ – (0.85108)).

One-way ANOVA of data sets (1) (Figure 4) revealed a high proportion of significantly altered features among the sample groups for cacao seeds in ESI (−) and CPH in ESI (+), in which more than 50% of the detected features showed statistically significant variation. For the seeds in ESI (+) and CPH in ESI (−), the value still ranged between 40 and 50%, comprising a substantial number of annotated features (Tables S3 and S4 **)**. Taking only the number of annotated features in each ionization mode and sample type into account, 55.80% in ESI (+) and 77.27% in ESI (−) for seeds were significant. For CPH, 59.22% in ESI (+) and 51.39% in ESI (−) were significant.

**Figure 4:**
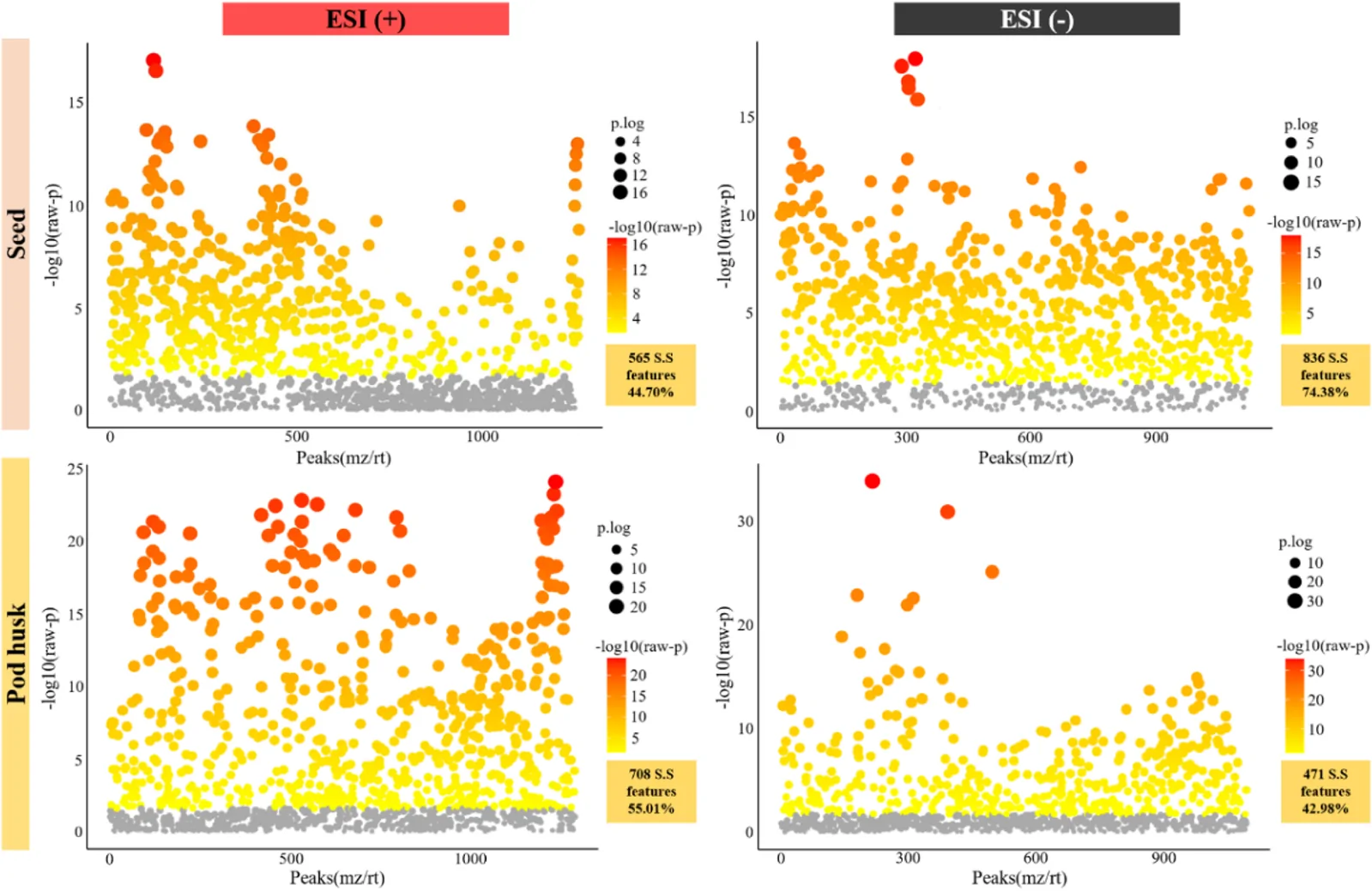
One-way ANOVA results for the different sample types in ESI (±) (*p* < 0.05).

### 3.3 Lipid Class Analysis

To obtain an integrated view of lipid remodeling during fruit maturation, class-level analysis was performed by summing the peak areas of all annotated lipids belonging to the same class in each sample. This approach provided an intermediate level of interpretation between the global lipidomic overview and the evaluation of individual lipid species, allowing the assessment of whether maturation-related changes were reflected in the overall abundance of functionally related lipid pools. A one-way ANOVA was performed in data sets (2), and a heatmap of significant classes with group clustering was generated to evaluate changes across maturation stages (Figure 5). Boxplots of the same classes and a heatmap without group clustering were constructed to visualize within-group variance (Figures S2 and S3).

**Figure 5:**
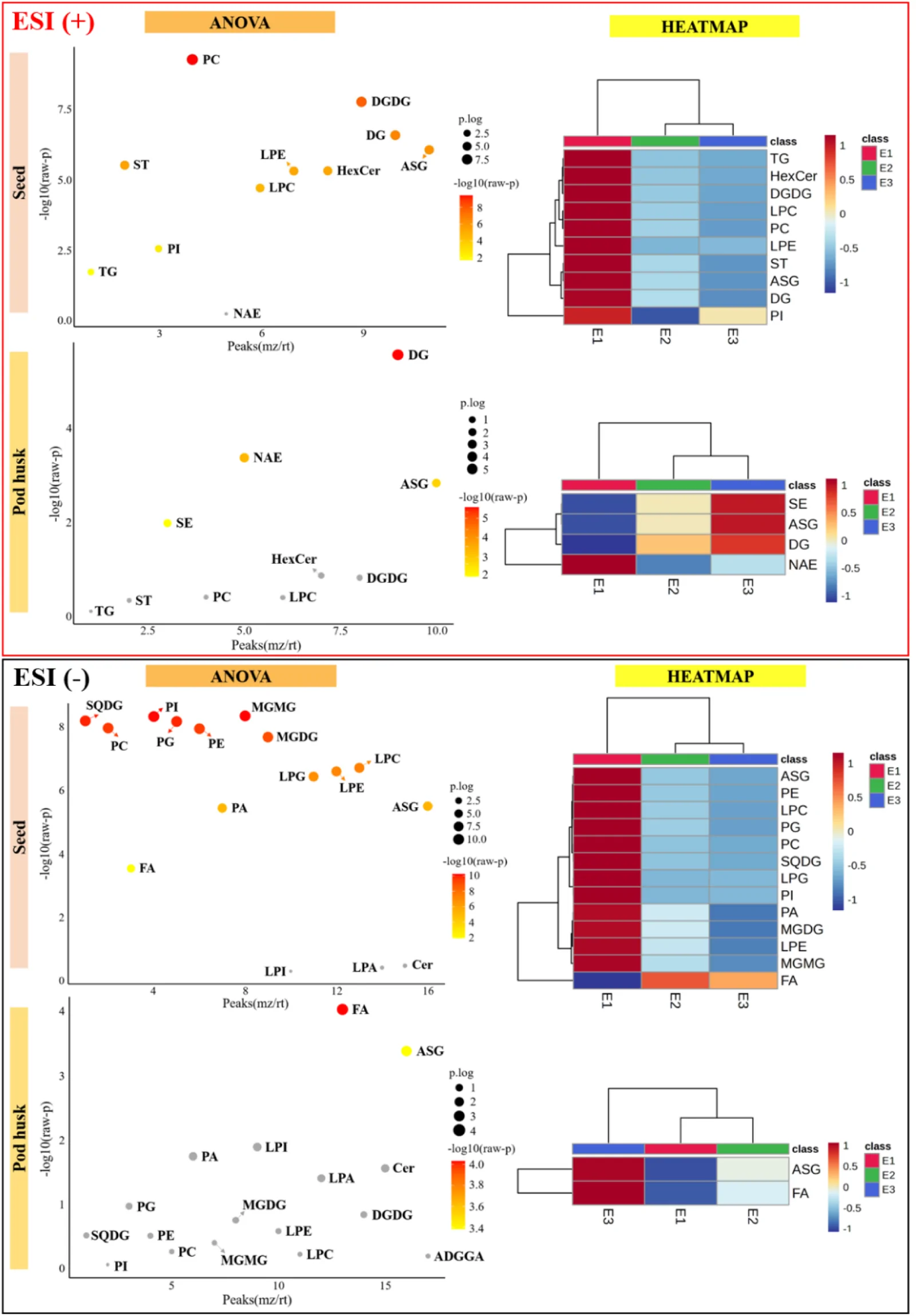
One-way ANOVA and heatmap of lipid classes for the different sample types in ESI (±) (*p* < 0.05).

It is important to note that significant variations between individual lipid-level (Table S4) and class-level (Figure 5) results were observed across the CPH data sets. In some cases, classes with significant annotated lipids did not change when evaluated as a whole. This may suggest that, in CPH, maturation promoted selective changes in specific annotated lipids without causing a coordinated shift in the overall abundance of the corresponding class. In addition, because class-level summed abundance includes both significant and nonsignificant annotated lipids, as well as lipids showing opposite trends during maturation, compensatory effects may have masked class-level variation.

Absolute lipid quantitation was not performed; therefore, no conclusions can be drawn regarding total lipid content. Rather, the present results support changes in the relative abundance of annotated lipid species and classes during seed maturation. Thus, these data are better interpreted as evidence of lipid remodeling across maturation stages than as a direct measure of total oil accumulation.

Consistent with this interpretation, Figure 5 and Table S4 show that in CPH, out of 13 lipid classes containing significant annotated lipids in ESI (−), only two were significant when assessed in the overall class-level matrix, and in ESI (+), four out of the 10 corresponding classes were significant. In addition, the lipid content of CPH has been reported to range from 1.5 to 6.87% on a dry-weight basis; however, details of lipid remodeling remain largely unexplored.^32,33^

The CPH (pericarp) consists of 67–77% of the cacao pod and is divided into three layers: epicarp (external), mesocarp (middle), and endocarp (inner), serving as the first barrier of protection against diseases and pests, being an indicator of the fruit’s health, with different functions.^32,34–37^ As for lipid composition, one study compared the tissues separately and with whole CPH as feed components in broiler chick diets, reporting higher crude fat content in the endocarp, followed by the epicarp and mesocarp, suggesting a distinct lipid distribution among the pericarp layers.^32,38^ Here, because CPH was analyzed as a whole, the heterogeneity of its layers and possible uneven changes across tissues may have influenced the overall class-level statistical results. Thus, in CPH, individual lipid analysis provides a more integrated view of some lipid changes.

Among statistically significant classes in both matrices, FA was the only group with lower intensity in E1, with differences between E2 and E3. Moreover, only one FA was a free fatty acid (FFA), FA 18:2, and the others were hydroxy FAs (HFA); therefore, the FA class here is mainly related to annotated HFAs, which showed the same overall trend as free FA 18:2 in the seed. This annotation pattern may also reflect the fact that FFAs are often rapidly incorporated into other lipid species, while their comprehensive analysis remains challenging due to poor ionization efficiency and the lack of characteristic fragment ions.^39–41^

Although HFAs are commonly associated with castor oil, they have been reported in plants. Sirbu et al. (2018) identified TGs with hydroxylated FAs in cocoa seeds, even though they are uncommon in seed oils.^42^ HFAs have been associated with plant self-defense substances and may intermediate oxidation pathways (mainly α- and β-oxidation).^42,43^

HFA has also been found in plants as constituents of sphingolipids in the plasma membrane (PM) and tonoplasts.^42^ In addition, these species have industrial importance and are used to make lubricants, motor oils, desiccants, nylon, plastics, cosmetics, pharmaceuticals, and polishes; therefore, further studies focusing on understanding their full constitution in cacao, especially CPH, can add value to this product that is commonly generated as waste.^10,42,44^

For other significant classes, only CPH contained SEs, which may be biomarkers of this tissue in PS1319. According to Figures 5 and S2, E1 had lower concentrations, increasing gradually to E2 and E3. This lipid functions as a storage form of sterols, accumulating in cytoplasmic lipid droplets (LDs) and contributing to the maintenance of PM sterol homeostasis throughout development.^45,46^

Sterols are important structural constituents of the PM, and both sterol deficiency and overaccumulation can impair plant growth and development. Among sterols, SEs are also a nontoxic form of sterol storage formed by excess free sterols (FS) conversion or carbon flux modulation in the pathway, as FS level regulation.^45,46^ The conversion happens through the transfer of a long-chain FA group to the free hydroxyl group of FS units using phospholipids (PL) and acyl-CoA donors. This reaction can be catalyzed by two sterol acyltransferases: phospholipid:sterol acyltransferase (PSAT) and acyl-CoA: sterol acyltransferase (ASAT).^46^

Therefore, the increase in SE may reflect enhanced sterol esterification, possibly associated with sterol buffering and membrane sterol homeostasis during maturation, especially in CPH, where a higher number of STs was observed.^45,46^ Similar cases have been reported for tomato maturation, where SEs increased significantly.

For ASG, a sterol conjugated with sugar and further acylated with a fatty acid, concentration increased during maturation in ESI (±), even with two ASG specific to ESI (+) and the other two for ESI (−), in contrast to seeds, where they decreased, suggesting a possible different sterol metabolism for the tissues.^46,47^

Regarding ASG biosynthesis, the activity of steryl glycoside acyltransferase has been detected, and partially purified enzyme preparations have been obtained, suggesting a complex scenario involving both the enzyme properties involved in biosynthesis and the nature of the acyl donors used. However, the genes encoding the enzyme have not been identified; therefore, the process is not fully elucidated.^48^ Concerning maturation, although the available evidence is limited and largely based on earlier studies, previous reports in tomato pericarp and bell pepper fruit suggest that ASGs may decrease during ripening, in contrast to findings in CPH and in accordance with the observed trend in cacao seeds, respectively.^49,50^

Finally, in CPH, DG increased intensity and variation throughout maturation, in contrast to the seeds. This response has been reported in peach mesocarp, where DG increased along with TG during maturation.^51^ They may be formed through *de novo* DG/TG synthesis (Kennedy pathway), as proposed in previous studies, in which FAs synthesis products in the plastid are exported and incorporated into this acyl-CoA-dependent pathway.^52^

The esterification of FAs to Coenzyme A (CoA), and the corresponding fatty acyl-CoA participating in glycerolipid synthesis, starts the synthesis, where sequential esterification to the *sn*-1 and *sn*-2 positions of glycerol-3-phosphate, catalyzed by acyl-CoA:glycerol-3-phosphate acyltransferase and acyl-CoA:lyso-phosphatidic acid acyltransferase, produces LPA followed by PA, respectively.^52^ The phosphate is removed by phosphatidic acid phosphatase, forming DG, which can be used for TG synthesis catalyzed by acyl-CoA:diacylglycerol acyltransferase (DGAT).^52,53^

As an intermediate of *de novo*, only one LPA species was detected in this matrix and did not change during maturation. However, LPAs are considered purely metabolic intermediates in the *de novo* pathway in plant cell membranes or for glycerophospholipid (GP) storage, which may explain the low number of annotations.^54^ For PAs, most species that showed significant variation decreased during maturation, suggesting that some may contribute to DG-related metabolism, and the observed increase. However, this cannot be confirmed without flux-based analysis. It is also possible that some DG species were used for TG synthesis, since a few species decreased intensity, but not strongly enough to affect DG and TG whole class relative abundance.^52,53^

In seeds, except for FAs and PIs in ESI (+), all classes with significant changes decreased in intensity across maturation. In the case of PI, this result only shows the trend for two species in this ionization mode; however, in ESI (−), where all PIs were annotated, the comprehensive analysis shows a decreasing trend.

One of the few studies on lipid changes during cacao maturation analyzed the total lipid content of Criollo cacao seeds of Ecuador with different postpollination days (125 – green, 170 – ripe). All species were extracted, separated into a few classes, and mostly quantified using thin-layer chromatography (TLC) and colorimetric tests, except for FAs that were analyzed by gas chromatography.^55^ They found that all lipid concentrations (DGs, FFAs, sterols, PLs, and glycolipids), except TGs, decreased during maturation, PLs especially; however, no analysis was performed to evaluate the statistical significance of these changes.^55^

Furthermore, most TG species did not change significantly (Table S3), and most of the significant features decreased during maturation. Notably, in seeds, three out of the 10 statistically significant TGs appeared to increase during maturation only after normalization, likely reflecting scaling effects driven by higher variability of peak intensities in E1. Before normalization, these three major cocoa butter TGs (TG 18:0/18:0/18:1 – SOS, TG 16:0/16:0/18:1 – POP, and TG 16:0/18:0/18:1 – POS) showed the trend E1 > E2 > E3, and normalized concentrations followed E2 > E3 > E1, showing significance only for the differences between E1 and E2 or E3 (Figure S4).^42^ These species are also visible in Figure 2 as the three most prominent peaks, at 7.85, 8.09, and 8.28 min. Thus, the major CB TGs appear to behave differently from several other annotated TG species, and their higher abundance in mature seeds may be associated with CB quality, particularly through their influence on melting behavior, crystallization, and texture.^42^

TGs can also be formed in the endoplasmic reticulum by an acyl-CoA-independent pathway, in addition to the dependent one.^53^ Without acyl-CoA, PLs act as acyl donors and DGs as acceptors, catalyzed by phospholipid:diacylglycerol acyltransferase (PDAT).^53^ A transcriptomic study on cacao seed development using DAP from two accessions suggested that the acyl-CoA-independent pathway might be the predominant one for TG synthesis in cacao seed.^53^ Finally, different responses were reported for PDAT genes, with an increasing trend being notable; however, the expression patterns shown for PDAT genes in different DAPs were not fully consistent across the reported figure, text, and tables, and their *p-*values were high (>0.1), limiting a strong interpretation of TG biosynthesis during maturation.^53^

In our study, because normalized peak intensities reflect relative abundance rather than absolute quantitation, the apparent decrease observed for many annotated TGs does not necessarily indicate a lower contribution of the major cocoa butter TGs to total seed oil. The principal TG species annotated possibly represent the dominant fraction of seed lipids, even though their normalized changes had limited influence on the overall class-level statistics; therefore, the results may not have a contrary response to what is expected of oilseeds.^42,56^ Moreover, the studied clone under the reported plantation conditions may also present a distinct maturation metabolism.^53,55,57^

It is also important to note that development is defined in two different “times”: developmental time, which is described by the sequence of stages, and chronological time, described by age, and both interact differently across species.^58^ Thus, differences can be observed when days after pollination (DAP) and different maturation stages are evaluated.^55,58^ However, similar responses were observed for lipid content from different chronological times and maturation stages, which is now being clarified in depth.

Finally, these results reveal a relevant aspect of cacao seed maturation, where annotated TG species did not behave uniformly across stages; rather than indicating a simple linear increase in TG accumulation, the data support selective remodeling of the TG profile during ripening. Energy storage mechanisms may influence this behavior in the oil bodies of these seeds, although this remains speculative in the absence of structural or imaging data.^59^

Concerning PLs, relative abundance decreased from E1 to E2 and E3. Great variation was observed within E1 for PCs, PAs, PGs, PEs, and their lyso forms, indicating greater biological variability. Similar results were also reported for cherry tomatoes, with most PLs decreased relative abundances at the final ripening stage.^61^ The metabolism of these lipids is essential for plant growth and development and relates to adaptability to environmental stress.^62^ Their biosynthesis is important for the formation of cellular membranes and involves various pathways to explain their presence in plants; however, the exact mechanism underlying their decreased concentration in ripe fruits remains unclear.^55,60–62^

In fruits, degradation of membrane PLs may be related to energy supply, as suggested in previous studies, although changes in these lipids are strongly linked to PL signaling, which is critical for plant development.^61–63^ This process involves the production of signaling molecules mediated by PLs through the action of phospholipases and lipid kinases, enzymes, and secondary messengers, with external responses mediation, and plant adaptation promotion.^61,63^

The phospholipases are classified based on the position PL is cleaved and the reaction products, which include A1, A2, C, and D, phospholipase A (PLA1 and PLA2), phospholipase C (PLC), and phospholipase D (PLD), respectively, and can present a preference for specific substrates.^63^ For example, phosphoinositide-specific phospholipase C hydrolyzes phosphatidylinositol 4,5-bisphosphate and produces inositol trisphosphate and DG, which can later participate in other reactions, forming PA that is critical to cellular regulation.^63^ PAs can also result from hydrolysis of PCs, PGs, and PEs via PLD activity.^63^

Considering PL metabolism, the changes observed, even if not explained in detail, are related to PL signaling, membrane lipid remodeling, and homeostasis.^61,63^ Finally, specific species within each PL class had different trends, although most decreased during maturation. This pattern suggests that a few individual PL species may have been involved in distinct metabolic pathways during seed maturation.

Concerning galactolipids (MGDG and DGDG) and SQDG, DGs formed in the *de novo* pathway can be used in their synthesis.^64^ MGDG and SQDG are formed from DG by MGDG synthase and a sulfolipid synthase, whereas DGDG results from the glycosylation of a small proportion of MGDG by DGDG synthase.^64^ As for the results, their relative abundance decreased across maturation, with no significant changes between E2 and E3 for SQDG, following the same pattern for DGDG.

These classes are mainly present in plant chloroplasts and are absent from most nonphotosynthetic organisms; however, they remain important constituents of the plastid membrane.^64–66^ Generally, in the early stages of development, fruits contain high levels of chlorophylls, and most plants undergo disintegration of the chloroplast grana and reduce photosynthetic activity during maturation.^67^ In 1993, a study considered that in developing oilseeds and nonphotosynthetic tissues, the proplastid was the sole site of *de novo* FA biosynthesis.^68^ At present, there is suggestive evidence of photosynthetic activity in seeds of oilseed rape and green oilseeds, which could indicate the presence of chloroplasts and their importance in these plants.^67^

On the other hand, there is no evidence demonstrating the presence of photosynthetically active chloroplasts in cacao seeds during fruit maturation. Therefore, the observed decrease in the intensity of these species may be more safely attributed to plastid-related membrane remodeling during maturation, since the presence and functional relevance of chloroplasts in cacao seeds have not yet been demonstrated.

In general, the annotated lipids underscore a constitution that may support additional applications for cacao byproducts, particularly CPH. PLs, TGs, and DGs are relevant to CB and chocolate quality, especially TGs.^12,69,70^ Although the overall relative abundance of most lipid classes was associated with green fruits, the major CB-related TGs, including SOS, POP, and POS, along with other specific lipids, showed higher abundance toward the mature stage, being relevant to CB quality.

Moreover, lipids are recognized as biorefinery feedstocks for food, health-related, and fuel-derived products.^71^ This perspective is relevant to CPH because its lipid fraction represents a form of carbon storage that may be useful for future valorization routes.^32,71^ By providing fatty acyl chains, lipids, especially TGs, contribute to biofuels and high-value chemicals production through catalytic pyrolysis.^72^ Studies have reported free FAs as important component of bio-oil obtained from sun-dried CPH pyrolysis, including linoleic acid as the predominant compound.^32^ Linoleic acid is also noteworthy because it has been investigated for skin-treatment applications, reinforcing the potential relevance of lipid-derived compounds.^32^ Furthermore, the annotation of HFAs in CPH emphasizes its potential for further industrial exploration, as previously mentioned. Thus, the diversity of lipids found in CPH expands the chemical description of this byproduct and highlights metabolites with technological interest.

Finally, these results reveal general lipid remodeling patterns, but their biochemical interpretation also depends on individual lipid species. For this reason, the main differential lipid biomarkers were further examined in detail.

### 3.4 Biomarkers

Based on the results presented in Figure 4, eight annotated features (Table S3) with the lowest *p*-values in the data set (2), as well as the most significant feature of each annotated class, with at least one significant feature, were selected for boxplot evaluations and discussions of maturation biomarkers. Figure 6 presents the eight most important annotated features, and Figure S5 shows the main annotated features of each class, both in cacao seeds in CPH.

**Figure 6:**
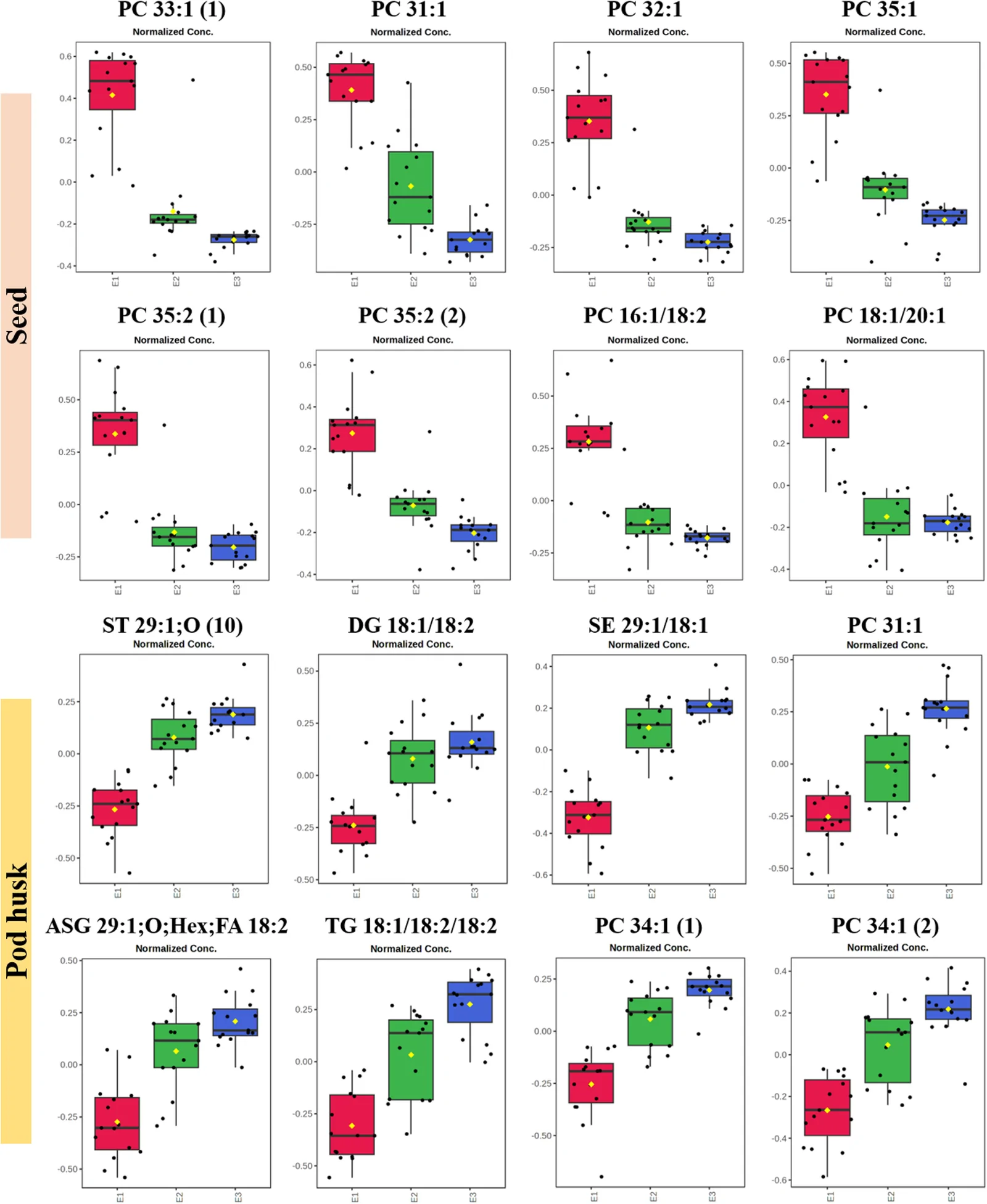
Boxplot of the eight features with the lowest *p*-values for ESI (+) in CPH and cacao seeds. Boxplot colors (red – E1; green – E2; blue – E3).

For cacao seeds, PCs were the most prominent among the features of Table S1, all following the same pattern of decreased intensity at more advanced maturation stages, and similar abundance between stages 2 and 3 (E2 and E3), with lower abundance commonly in E3. In addition, post hoc tests showed differences between E1 and E2 or E3 in most cases. As presented in Section 3.2, studies on cacao show a decrease in phospholipids during maturation with different DAPs, following the same pattern in developmental time analysis.^55,58^

In the case of cacao seeds in Figure S5, for most classes, the main lipid is more intense in green fruits, with a gradual decrease in normalized concentration from the first stage to the others. Here, the annotated lipids may have appeared in all samples, but higher concentrations for most annotated species usually indicate green fruit conditions.

In CPH, although overall class abundance did not change significantly in most cases, some species showed distinct trends compared to their counterparts in seeds. Except for the HexCer, NAE, and LPC examples, the prominent features of each class increased during maturation, marking intermediate and ripe fruits as representatives. Studies on lipid profile changes during the maturation of fruit pericarp are limited, hindering a more in-depth discussion of specific lipid increase, particularly when overall classes’ abundance indicates no significant changes, possibly due to opposing trends among species within the same class.

In the context of lipid species within the same class exhibiting opposing trends and masking overall class-level changes, a general redistribution among lipid species, along with membrane remodeling, is proposed as an explanation; however, this underscores the need for further targeted studies.^56^ The results also show that some changes are opposite when comparing seeds and CPH.

Finally, NAEs in seeds did not change, and only one species changed in CPH; however, its presence in plants remains poorly documented.^73,74^ NAEs are important for plant growth and function as signaling lipids. Even small amounts of these compounds may be required throughout maturation, thereby continuing with a relatively stable composition.^73,74^

Moreover, for ESI (−), Figures 7 and S6 show the eight most important annotated features and the feature with the lowest *p*-value in each class boxplot. Observed trends are similar to ESI (+), and PLs predominantly differentiate the maturation stages, indicating that green fruits are the main group. In CPH, opposite patterns are observed.

**Figure 7:**
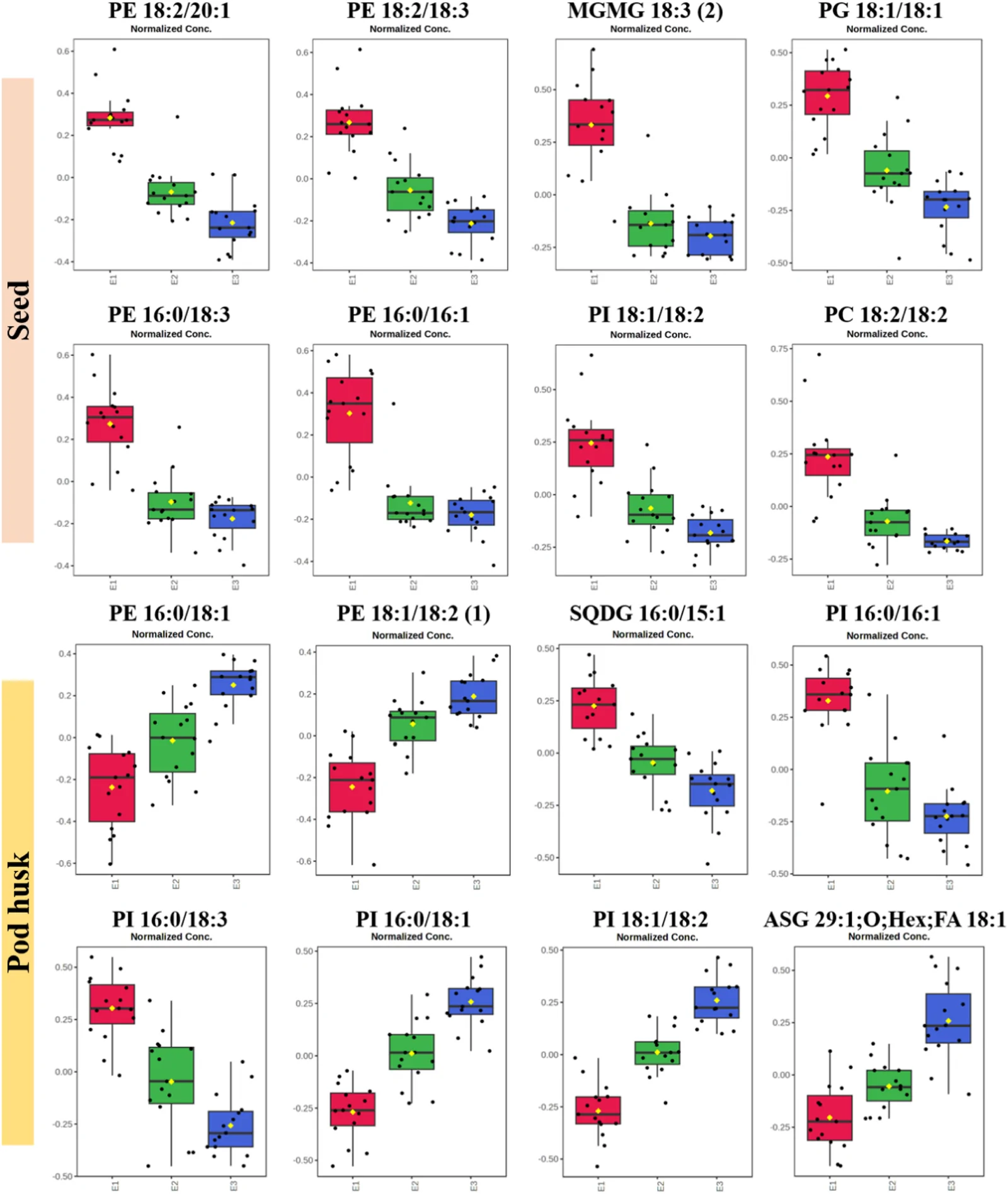
Boxplot of the eight features with the lowest *p*-values for ESI (−) in CPH and cacao seeds. Boxplot colors (red – E1; green – E2; blue – E3).

According to Figure 7, prominent features and most species in the same classes in seeds are biomarkers of E1. In CPH, biomarkers of different maturation stages within the same class appear, with five features increasing during ripening and four decreasing. This opposing pattern likely explains why many classes did not show statistically significant changes. Furthermore, it reinforces the idea that membrane remodeling may have occurred in a species-specific manner, without substantially affecting the overall class in this matrix, although this hypothesis requires further validation.

In Figure S6, although all classes, except FA, decreased in data set (2), the most significant MGDG, LPI, and LPA species increased, and are markers of intermediate and ripe fruits. For CPH, more than half of these features decreased, showing E1 as the main stage to find them, whereas for those increasing, it is possible to see that for most, E2 and E3 are statistically the same; however, PI 18:1/18:2 showed an important difference among the stages, being a biomarker of ripe CPH.

Overall, in seeds, a higher abundance of most features indicates that the sample corresponds to the green stage, whereas annotated features increasing during maturation tend to be statistically equal between intermediate and ripe fruits. Among the most statistically significant species selected within each lipid class, only FA, MGDG, LPI, LPA, and Cer included markers associated with E2 and/or E3; thus, most lipids with more pronounced statistical differences were mainly useful for identifying green-stage seed samples and distinguishing them from later developmental stages. On the other hand, CPH showed greater variation in lipid trends within the same class, with some species acting as markers of E1, such as LPC 18:0, PG 16:0/15:1, and LPE 20:0, among others, and others associated with E3, such as PC 34:1, TG 18:1/18:2/18:2, and FA 26:0­(2OH), among others. This tissue-specific behavior indicates that seed lipid markers mainly reflect the transition from green to later developmental stages, whereas CPH lipid markers provide complementary information on pericarp maturation and chemical differentiation of this byproduct.

These lipid markers provide analytical guidance on harvest timing by showing that green seeds have a distinct lipid profile from later developmental stages. Since seeds and CPH were obtained from fruits classified within the same maturation stages, the presence of E3-associated markers in CPH indicates that the pericarp also carries lipid signatures of fruit ripeness. Thus, lipid profiling of CPH may provide complementary analytical information for identifying ripe fruits. Collectively, these findings show that cacao maturation involves broad lipid remodeling, with class-level trends providing an integrated view and biomarker-level analysis revealing the molecular specificity underlying these changes.

## Supplementary Material



## Notes

Additional experimental details and data processing parameters, including ESI source, MS-DIAL, and MetaboAnalyst parameters; supplementary results, including annotated lipid tables for cacao seeds and cacao pod husk with adducts, *m*/*z* values, retention times, and one-way ANOVA *p*-values; QC RSD assessment; additional boxplots; and heatmaps (PDF)
