# Supplementary material for: From Green to Ripe: Untargeted UHPLC–Orbitrap–MS Lipidomic Profiling of Cacao Seed and Pod Husk during Maturation
Source: J Agric Food Chem. 2026 Aug 29;74(35):28039–52. doi: 10.1021/acs.jafc.6c08470 (PMC13573563; doi:10.1021/acs.jafc.6c08470)
Supplement: Supplementary file 1 [file jf6c08470_si_001.pdf]

From Green to Ripe: Untargeted UHPLC–Orbitrap–MS Lipidomic Profiling of Cacao Seed and Pod Husk During Maturation

João V. M. de Almeida[a], Daniel S. Trancoso[b], Eclair V. Filho[b], Alan R. Pereira[a], Anna C. F. Braz[c], Nathália dos S. Conceição[a], Julie M. M. Figueiredo[c], Marcio V. Rodrigues[d], Nayara A. dos Santos[e], Ricardo M. Kuster[a], Wanderson Romão[a,b,c*]

[a]Federal University of Espírito Santo/UFES, Department of Chemistry, Campus Goiabeiras, Avenida Fernando Ferrari, 514, 29075-910 Vitoria, ES, Brazil.

[b]Federal University of Espírito Santo/UFES, Department of Pharmaceutical Sciences, Campus Maruipe, Avenida Marechal Campos, 1468, 29043-213 Vitoria, ES, Brazil.

[c]Federal Institute of Espírito Santo/IFES, Campus Vila Velha, Av. Ministro Salgado Filho, 29106-010, Vila Velha, ES, Brazil.

[d]Federal Institute of Espírito Santo/IFES, Campus Linhares, Av. Filogônio Peixoto, 29901-291, Linhares, ES, Brazil.

[e]Federal Institute of Espírito Santo/IFES, Campus Vitória, Av. Vitória, 29040-780, Vitória, ES, Brazil.

Correspondence should be addressed to: Wanderson Romão (wanderson.romao@ifes.edu.br)

Table S1. ESI parameters.

Ion transfer tube temperature	Vaporizer temperature	Sheath gas	Auxiliary gas	Sweep gas	Source voltage
320 °C	350 °C	50 AU	10 AU	1 AU	3.5 kV (+) and 2.5 kV (-)

AU – arbitrary units.

Table S2. MS-DIAL parameters.

Sample / Ionization mode	Peak detection				Spectrum deconvolution	Alignment parameter			
	M.P.H	M.S.W	S.L	M.P.W	S.W.V	Reference	Rt tolerance	B.F-Sa.Bla.	N% group
Seeds / ESI (+)	2.0E6	5.0E-2	3.0	5.0	0.5	QC5	0.1	10 fold	60
Seeds / ESI (-)	5.0E5	5.0E-2	3.0	5.0	0.5	QC5	0.1	10 fold	60
CPH / ESI (+)	2.0E6	5.0E-2	3.0	5.0	0.5	QC3	0.1	10 fold	60
CPH / ESI (-)	5.0E5	5.0E-2	3.0	5.0	0.5	QC3	0.1	10 fold	60

M.P.H – Minimum peak height; M.S.W – Mass slice width; S.L – Smoothing level; M.P.W – Minimum peak width; S.W.V – Sigma window value; Rt – Retention time; CPH – cacao pod husk; B.F-Sa.Bla – Blank filtering – sample average/blank average; N% group – N% detected in at least one group.

Table S3. MetaboAnalyst normalization parameters.

Sample	Dataset/ionization mode	Sample normalization	Data transformation	Data scaling
Seeds	(1) / ESI (+)	Sum	Log_2	Range
	(2) / ESI (+)	None	Log_2	Pareto
	(1) / ESI (-)	Sum	Log_{10}	Range
	(2) / ESI (-)	None	Log_2	Pareto
CPH	(1) / ESI (+)	Sum	Log_{10}	Range
	(2) / ESI (+)	None	Log_{10}	Pareto
	(1) / ESI (-)	Sum	Log_{10}	Range
	(2) / ESI (-)	None	Log_2	Range

CPH – cacao pod husk. (1) – dataset of all features with MS/MS; (2) – dataset of sums within classes.

Table S4. Annotated lipids in cacao seeds with ionization mode-specific ANOVA results.

Name	Adduct	*m/z*	Rt(min)	*p*-value (ANOVA)
TG 16:0/15:1/18:2	$[M + NH_4]^+$	832.7369	7.10	1.14E-03
TG 18:0/18:1/18:1	$[M + NH_4]^+$	904.8305	7.18	5.21E-03
TG 16:0/16:1/16:1	$[M + NH_4]^+$	820.7368	7.17	1.04E-03
TG 16:0/16:1/18:2	$[M + NH_4]^+$	846.7523	7.24	1.62E-06
TG 16:0/18:2/18:2	$[M + NH_4]^+$	872.7674	7.26	>0.05 (SI)
TG 18:1/18:1/18:3	$[M + NH_4]^+$	898.7830	7.27	>0.05 (SI)
TG 15:0/16:0/18:2	$[M + NH_4]^+$	834.7527	7.38	1.07E-04
TG 17:0/18:2/18:2	$[M + NH_4]^+$	886.7838	7.44	>0.05 (SI)
TG 14:0/16:0/18:1	$[M + NH_4]^+$	822.7526	7.52	>0.05 (SI)
TG 16:0/16:0/18:2	$[M + NH_4]^+$	848.7680	7.56	2.33E-07
TG 16:0/18:1/18:2 (1)	$[M + NH_4]^+$	874.7831	7.58	>0.05 (SI)
TG 18:1/18:1/18:2	$[M + NH_4]^+$	900.7990	7.63	>0.05 (SI)
TG 15:0/16:0/18:1	$[M + NH_4]^+$	836.7681	7.70	>0.05 (SI)
TG 16:0/17:2/18:1	$[M + NH_4]^+$	862.7835	7.73	8.25E-06
TG 16:0/18:1/18:2 (2)	$[M + NH_4]^+$	874.7831	7.80	>0.05 (SI)
TG 16:0/16:0/16:0	$[M + NH_4]^+$	824.7686	7.83	>0.05 (SI)
TG 16:0/16:0/18:1	$[M + NH_4]^+$	850.7834	7.85	1.84E-03
TG 18:0/16:1/18:1	$[M + NH_4]^+$	876.7990	7.89	>0.05 (SI)
TG 18:1/18:1/18:1	$[M + NH_4]^+$	902.8145	7.91	>0.05 (SI)
TG 20:0/18:2/18:2	$[M + NH_4]^+$	928.8303	7.94	>0.05 (SI)

TG 16:0/17:0/18:1	$[M + NH_4]^+$	864.7996	7.99	>0.05 (SI)
TG 18:0/17:1/18:1	$[M + NH_4]^+$	890.8149	8.01	>0.05 (SI)
TG 16:0/16:0/18:0	$[M + NH_4]^+$	852.8001	8.08	>0.05 (SI)
TG 16:0/18:0/18:1	$[M + NH_4]^+$	878.8150	8.09	1.88E-04
TG 20:0/18:1/18:2	$[M + NH_4]^+$	930.8458	8.15	>0.05 (SI)
TG 22:0/18:2/18:2	$[M + NH_4]^+$	956.8621	8.16	>0.05 (SI)
TG 16:0/17:0/18:0	$[M + NH_4]^+$	866.8156	8.19	>0.05 (SI)
TG 17:0/18:0/18:1	$[M + NH_4]^+$	892.8307	8.20	>0.05 (SI)
TG 21:0/18:1/18:2	$[M + NH_4]^+$	944.8620	8.23	>0.05 (SI)
TG 16:0/18:0/18:0	$[M + NH_4]^+$	880.8308	8.26	>0.05 (SI)
TG 18:0/18:0/18:1	$[M + NH_4]^+$	906.8466	8.28	2.02E-03
TG 20:0/18:1/18:1	$[M + NH_4]^+$	932.8614	8.30	>0.05 (SI)
TG 22:0/18:1/18:2	$[M + NH_4]^+$	958.8773	8.33	>0.05 (SI)
TG 24:0/18:2/18:2	$[M + NH_4]^+$	984.8932	8.33	>0.05 (SI)
TG 17:0/18:0/18:0	$[M + NH_4]^+$	894.8473	8.36	>0.05 (SI)
TG 23:0/16:1/18:1	$[M + NH_4]^+$	946.8777	8.38	>0.05 (SI)
TG 18:0/18:0/18:0	$[M + NH_4]^+$	908.8624	8.43	>0.05 (SI)
TG 18:0/20:0/18:1	$[M + NH_4]^+$	934.8779	8.44	>0.05 (SI)
TG 18:0/18:1/22:1	$[M + NH_4]^+$	960.8931	8.46	>0.05 (SI)
TG 24:0/18:1/18:2	$[M + NH_4]^+$	989.9090	8.47	>0.05 (SI)
TG 16:0/18:0/21:0	$[M + NH_4]^+$	922.8782	8.51	>0.05 (SI)
TG 23:0/18:1/18:1	$[M + NH_4]^+$	974.9090	8.53	>0.05 (SI)

TG 16:0/18:0/22:0	$[M + NH_4]^+$	936.8939	8.59	>0.05 (SI)
TG 18:0/22:0/18:1	$[M + NH_4]^+$	962.9091	8.60	>0.05 (SI)
TG 18:0/18:1/24:1	$[M + NH_4]^+$	988.9244	8.61	>0.05 (SI)
TG 26:0/18:1/18:2	$[M + NH_4]^+$	1014.9402	8.62	>0.05 (SI)
TG 16:0/18:0/23:0	$[M + NH_4]^+$	950.9099	8.66	>0.05 (SI)
TG 18:0/23:0/18:1	$[M + NH_4]^+$	976.9249	8.67	>0.05 (SI)
TG 25:0/18:1/18:1	$[M + NH_4]^+$	1002.9407	8.68	>0.05 (SI)
TG 16:0/18:0/24:0	$[M + NH_4]^+$	964.9252	8.74	>0.05 (SI)
TG 18:0/24:0/18:1	$[M + NH_4]^+$	990.9403	8.74	>0.05 (SI)
TG 26:0/18:1/18:1	$[M + NH_4]^+$	1016.9563	8.75	>0.05 (SI)
TG 18:0/18:0/24:0	$[M + NH_4]^+$	992.9565	8.88	>0.05 (SI)
TG 18:0/25:0/18:1	$[M + NH_4]^+$	1004.9565	8.82	>0.05 (SI)
TG 18:0/26:0/18:1	$[M + NH_4]^+$	1018.9724	8.89	>0.05 (SI)
TG 18:0/28:0/18:2	$[M + NH_4]^+$	1044.9879	8.90	>0.05 (SI)
TG 18:0/27:0/18:1	$[M + NH_4]^+$	1032.9884	8.96	>0.05 (SI)
TG 18:0/28:0/18:1	$[M + NH_4]^+$	1047.0033	9.04	>0.05 (SI)
ST 29:1;O	$[M + H - H_2O]^+$	397.3824	5.96	1.56E-04
PI 18:0/18:1	$[M + Na]^+$	887.5601	4.69	>0.05 (SI)
	$[M - H]^-$	863.5650	4.70	>0.05 (SI)
PI 16:0/18:2	$[M + Na]^+$	857.5131	4.49	>0.05 (SI)
	$[M - H]^-$	833.5182	4.49	>0.05 (SI)
PC 18:0/20:3	$[M + H]^+$	812.6141	5.05	1.66E-06

PC 40:2	$[M + H]^+$	842.6618	5.60	1.48E-07
PC 18:1/20:1	$[M + H]^+$	814.6306	5.21	5.04E-11
PC 38:2	$[M + H]^+$	814.6304	5.36	4.66E-06
	$[M + HCOO]^-$	858.6233	5.25	4.48E-08
PC 37:2	$[M + H]^+$	800.6152	5.26	2.15E-08
PC 37:1	$[M + H]^+$	802.6308	5.44	5.87E-12
PC 36:5	$[M + H]^+$	780.5518	4.64	3.26E-10
PC 36:4	$[M + H]^+$	782.5672	4.75	3.57E-10
	$[M + HCOO]^-$	826.5601	4.76	2.34E-09
PC 16:0/18:1	$[M + H]^+$	782.5656	5.03	3.18E-05
	$[M + Na]^+$	806.5648	4.88	7.17E-08
PC 36:3	$[M + H]^+$	784.5825	4.89	2.30E-09
	$[M + HCOO]^-$	828.5759	4.89	4.93E-08
	$[M + Na]^+$	808.5811	5.03	2.61E-07
PC 18:1/18:1	$[M + H]^+$	786.5982	5.03	1.70E-07
	$[M + HCOO]^-$	830.5916	5.03	1.99E-06
	$[M + H]^+$	788.6146	5.22	1.71E-08
PC 18:0/18:1	$[M + Na]^+$	810.597	5.23	2.05E-08
	$[M + HCOO]^-$	832.6078	5.25	4.66E-08
PC 35:7	$[M + H]^+$	762.5031	4.66	4.44E-09
PC 17:1/18:2	$[M + H]^+$	770.5679	4.82	4.20E-08
PC 35:2 (1)	$[M + H]^+$	772.5833	4.82	3.58E-11

PC 35:2 (2)	$[M + H]^+$	772.5835	5.08	1.97E-11
PC 35:1	$[M + H]^+$	774.5997	5.22	1.01E-12
PC 34:3	$[M + H]^+$	756.5518	4.75	4.53E-11
	$[M + HCOO]^-$	800.5445	4.76	1.05E-09
PC 16:0/18:2	$[M + H]^+$	758.5672	4.88	1.21E-10
PC 34:1	$[M + H]^+$	760.5831	5.03	1.65E-05
PC 18:1/16:0	$[M + H]^+$	760.5835	5.19	3.94E-04
PC 33:3	$[M + H]^+$	742.5364	4.77	1.38E-07
PC 33:2	$[M + H]^+$	744.5526	4.89	1.63E-09
PC 33:1	$[M + H]^+$	746.5687	5.04	3.83E-14
PC 15:2/18:2	$[M + H]^+$	740.5213	4.66	9.86E-09
PC 33:5	$[M + H]^+$	738.5056	4.57	2.10E-11
PC 32:2	$[M + H]^+$	730.5368	4.72	3.54E-10
PC 32:1	$[M + H]^+$	732.5523	4.85	1.29E-13
PC 31:2	$[M + H]^+$	716.5209	4.75	1.62E-08
PC 31:1	$[M + H]^+$	718.5368	4.87	6.63E-14
PC 31:4	$[M + H]^+$	712.4901	4.53	3.29E-07
PC 31:3	$[M + H]^+$	714.5061	4.34	2.60E-03
NAE 4:0	$[M + H]^+$	132.1017	0.90	1.41E-05
NAE 20:2	$[M + H]^+$	352.3206	4.15	>0.05 (SI)
NAE 18:2	$[M + H]^+$	324.2893	4.14	>0.05 (SI)
NAE 18:1	$[M + H]^+$	326.3050	4.27	>0.05 (SI)

NAE 14:0	$[M + H]^+$	272.2583	2.26	>0.05 (SI)
LPC 18:2 (1)	$[M + HCOO]^-$	564.3307	3.66	>0.05 (SI)
	$[M + H]^+$	520.3391	3.65	>0.05 (SI)
LPC 18:2 (2)	$[M + HCOO]^-$	564.3309	3.47	4.42E-10
	$[M + H]^+$	520.3390	3.45	1.33E-09
LPC 18:1 (1)	$[M + H]^+$	522.3547	4.02	>0.05 (SI)
	$[M + HCOO]^-$	566.3464	4.02	>0.05 (SI)
LPC 18:1 (2)	$[M + H]^+$	522.3546	3.96	7.86E-08
	$[M + HCOO]^-$	566.3461	3.96	2.27E-07
LPC 16:0	$[M + H]^+$	493.3392	3.93	3.54E-03
HexCer 18:2;2O/24:0;O	$[M + H]^+$	826.6753	5.24	1.07E-04
HexCer 18:2;2O/20:1	$[M + H]^+$	752.6013	4.90	6.60E-05
HexCer 18:2;2O/16:1	$[M + H]^+$	696.5396	4.62	1.92E-03
HexCer 18:2;2O/16:0	$[M + H]^+$	698.5557	4.67	3.14E-05
LPE 18:1	$[M + H]^+$	480.3078	3.96	2.35E-05
	$[M - H]^-$	478.2935	3.99	2.68E-03
DGDG 18:3/18:3	$[M + NH_4]^+$	954.6127	4.41	2.93E-08
DGDG 18:1/18:3	$[M + NH_4]^+$	958.6440	4.57	1.90E-06
DGDG 18:0/18:3	$[M + NH_4]^+$	960.6599	4.67	3.24E-06
DGDG 18:0/18:2	$[M + NH_4]^+$	962.6754	4.77	>0.05 (SI)
DGDG 18:0/18:1	$[M + NH_4]^+$	964.6914	4.90	>0.05 (SI)
DGDG 16:0/18:3	$[M + NH_4]^+$	932.6284	4.56	1.10E-10

DGDG 16:0/18:2	$[M + NH_4]^+$	934.6439	4.65	1.30E-05
DGDG 16:0/18:1	$[M + NH_4]^+$	936.6596	4.76	>0.05 (SI)
DG 20:0/18:1	$[M + NH_4]^+$	668.6182	5.86	5.94E-06
DG 18:1/18:1	$[M + NH_4]^+$	638.5706	5.37	2.43E-08
	$[M + Na]^+$	643.5262	5.37	3.30E-05
DG 18:0/18:1	$[M + NH_4]^+$	640.5864	5.58	1.39E-04
DG 18:0/18:0	$[M + NH_4]^+$	642.6026	5.84	2.38E-05
DG 17:0/18:1	$[M + NH_4]^+$	626.5713	5.45	9.90E-07
DG 16:0/18:1	$[M + NH_4]^+$	612.5551	5.35	4.70E-07
DG 16:0/16:1	$[M + NH_4]^+$	584.5242	5.14	3.67E-06
DG 20:0/18:2	$[M + NH_4]^+$	666.6020	5.61	3.26E-03
DG 18:2/18:3	$[M + NH_4]^+$	632.5239	4.90	9.45E-03
DG 18:1/18:2	$[M + NH_4]^+$	636.5554	5.18	>0.05 (SI)
DG 16:0/18:2	$[M + NH_4]^+$	610.5397	5.17	>0.05 (SI)
ASG 29:1;O;Hex;FA 18:1	$[M + NH_4]^+$	858.7162	5.97	7.74E-08
	$[M + HCOO]^-$	885.6825	5.97	>0.05 (SI)
ASG 29:1;O;Hex;FA 18:0	$[M + NH_4]^+$	860.7324	6.27	1.00E-02
	$[M + HCOO]^-$	887.6981	6.27	2.79E-05
ASG 29:1;O;Hex;FA 16:0	$[M + NH_4]^+$	832.7007	5.95	1.00E-02
	$[M + HCOO]^-$	859.6673	5.95	>0.05 (SI)
ASG 28:1;O;Hex;FA 16:0	$[M + NH_4]^+$	818.6854	5.85	2.00E-02
	$[M + HCOO]^-$	845.6518	5.85	>0.05 (SI)

SQDG 36:6	$[M - H]^-$	837.4833	4.26	3.21E-02
SQDG 18:2/18:3	$[M - H]^-$	839.4990	4.31	1.17E-07
SQDG 18:1/18:3	$[M - H]^-$	841.5142	4.37	5.80E-03
SQDG 36:3	$[M - H]^-$	843.5298	4.45	7.52E-03
SQDG 36:2	$[M - H]^-$	845.5449	4.52	>0.05 (SI)
SQDG 18:0/18:1	$[M - H]^-$	847.5607	4.60	>0.05 (SI)
SQDG 34:3	$[M - H]^-$	815.4986	4.37	4.17E-05
SQDG 34:2	$[M - H]^-$	817.5144	4.43	3.55E-03
SQDG 16:0/16:0	$[M - H]^-$	793.5142	4.50	>0.05 (SI)
SQDG 16:0/16:1	$[M - H]^-$	791.4969	4.44	2.28E-08
FA 22:0(2OH) (1)	$[M - H]^-$	355.3214	5.35	1.93E-02
FA 26:0(2OH) (1)	$[M - H]^-$	411.3840	4.99	1.72E-07
FA 26:0(2OH) (2)	$[M - H]^-$	411.3839	5.18	1.48E-06
FA 25:0(2OH)	$[M - H]^-$	397.3682	4.91	8.50E-08
FA 24:0(2OH) (1)	$[M - H]^-$	383.3528	5.35	3.16E-04
FA 24:0(2OH) (2)	$[M - H]^-$	383.3528	5.18	2.43E-05
FA 24:0(2OH) (3)	$[M - H]^-$	383.3525	4.82	8.56E-07
FA 23:0(2OH)	$[M - H]^-$	369.3368	4.74	9.72E-08
FA 22:0(2OH) (2)	$[M - H]^-$	355.3214	5.18	3.39E-03
FA 22:0(2OH) (3)	$[M - H]^-$	355.3211	4.66	2.81E-04
FA 21:0(2OH)	$[M - H]^-$	341.3056	4.60	6.28E-06
FA 20:0(2OH)	$[M - H]^-$	327.2904	4.53	1.43E-02

FA 18:2	$[M - H]^-$	279.2330	4.08	3.43E-06
FA 18:0(2OH)	$[M - H]^-$	299.2592	4.42	>0.05 (SI)
FA 16:0(2OH)	$[M - H]^-$	271.2279	4.29	9.70E-03
PI 18:2/18:3	$[M - H]^-$	855.5027	4.36	1.16E-03
PI 18:2/18:2	$[M - H]^-$	857.5181	4.43	5.35E-05
PI 18:1/18:2	$[M - H]^-$	859.5336	4.50	1.34E-09
PI 36:2	$[M - H]^-$	861.5491	4.60	1.21E-06
PI 17:0/18:2	$[M - H]^-$	847.5336	4.55	2.20E-06
PI 17:0/18:1	$[M - H]^-$	849.5491	4.64	>0.05 (SI)
PI 16:0/18:3	$[M - H]^-$	831.5030	4.43	3.73E-09
PI 16:0/18:2 (2)	$[M - H]^-$	833.5185	4.60	>0.05 (SI)
PI 16:0/18:2;O	$[M - H]^-$	849.5134	4.22	>0.05 (SI)
PI 16:0/18:1 (1)	$[M - H]^-$	835.5347	4.98	>0.05 (SI)
PI 16:0/18:1 (2)	$[M - H]^-$	835.5347	5.18	1.93E-05
PI 16:0/18:1 (3)	$[M - H]^-$	835.5339	4.59	>0.05 (SI)
PI 14:0/18:2	$[M - H]^-$	805.4872	4.40	7.93E-03
PI 16:0/16:1	$[M - H]^-$	807.5026	4.47	1.22E-05
PG 46:1	$[M - H]^-$	915.7064	5.18	1.84E-02
PG 18:4/16:0	$[M - H]^-$	741.4689	4.84	1.76E-05
PG 18:2/18:2 (1)	$[M - H]^-$	769.5024	4.84	2.17E-04
PG 18:2/18:2 (2)	$[M - H]^-$	769.5024	4.47	>0.05 (SI)
PG 18:1/18:2	$[M - H]^-$	771.5180	4.54	>0.05 (SI)

PG 18:1/18:1	$[M - H]^-$	773.5341	4.62	5.22E-11
PG 18:0/18:1	$[M - H]^-$	775.5494	4.76	9.39E-08
PG 16:0/18:3	$[M - H]^-$	743.4872	4.46	7.34E-09
PG 16:0/18:2	$[M - H]^-$	745.5026	4.54	5.87E-04
PG 16:0/18:1	$[M - H]^-$	747.5186	4.63	5.48E-09
PG 16:0/18:0	$[M - H]^-$	749.5337	4.74	2.11E-05
PG 16:0/16:1	$[M - H]^-$	719.4871	4.52	3.74E-04
PG 16:0/16:0	$[M - H]^-$	721.5025	4.61	6.33E-05
PE 28:0/18:2	$[M - H]^-$	882.6965	6.06	1.16E-04
PE 26:0/18:2	$[M - H]^-$	854.6651	5.76	5.79E-08
PE 26:0/18:1	$[M - H]^-$	856.6805	6.00	6.94E-06
PE 25:0/18:2	$[M - H]^-$	840.6492	5.62	3.31E-07
PE 25:0/18:1	$[M - H]^-$	842.6648	5.85	1.46E-03
PE 24:0/18:2	$[M - H]^-$	826.6333	5.50	2.63E-09
PE 24:0/18:1	$[M - H]^-$	828.6493	5.70	4.30E-08
PE 23:0/18:1	$[M - H]^-$	814.6335	5.57	1.76E-04
PE 23:0/18:2	$[M - H]^-$	812.6178	5.38	2.98E-06
PE 22:0/18:3	$[M - H]^-$	796.5866	5.13	1.09E-02
PE 22:0/18:2	$[M - H]^-$	798.6018	5.27	5.79E-08
PE 22:0/18:1	$[M - H]^-$	800.6180	5.45	2.39E-06
PE 18:2/18:3	$[M - H]^-$	736.4926	4.57	4.70E-12
PE 18:2/18:2 (1)	$[M - H]^-$	738.5084	4.41	4.57E-05

PE 18:2/18:2 (2)	$[M - H]^-$	738.5079	4.67	2.15E-09
PE 18:2/18:2;O	$[M - H]^-$	754.5030	4.31	1.62E-02
PE 18:1/18:2	$[M - H]^-$	740.5235	4.78	2.77E-06
PE 16:0/18:3	$[M - H]^-$	712.4922	4.66	6.04E-11
PE 16:0/18:2 (1)	$[M - H]^-$	714.5083	5.15	4.06E-05
PE 16:0/18:2 (2)	$[M - H]^-$	714.5078	4.76	5.39E-08
PE 16:0/18:1	$[M - H]^-$	716.5233	4.88	>0.05 (SI)
PE 16:0/16:1	$[M - H]^-$	688.4924	4.74	1.02E-10
PE 18:1/20:0	$[M - H]^-$	772.5864	5.23	2.03E-07
PE 18:1/20:1	$[M - H]^-$	770.5706	5.07	3.08E-10
PE 18:0/18:1	$[M - H]^-$	744.5551	5.04	7.51E-08
PE 18:2/20:1	$[M - H]^-$	768.5549	4.91	3.69E-13
PE 36:2	$[M - H]^-$	742.5393	4.90	4.12E-07
PE 18:1/18:1	$[M - H]^-$	742.5394	4.55	1.30E-03
PA 22:0/18:2	$[M - H]^-$	755.5601	5.35	>0.05 (SI)
PA 22:0/18:1	$[M - H]^-$	757.5757	5.58	3.44E-06
PA 20:0/18:2	$[M - H]^-$	727.5291	5.09	>0.05 (SI)
PA 20:0/18:1	$[M - H]^-$	729.5445	5.32	6.52E-04
PA 18:1/19:2	$[M - H]^-$	711.4972	4.82	1.68E-03
PA 18:2/18:3	$[M - H]^-$	693.4504	4.56	6.52E-05
PA 18:2/18:2	$[M - H]^-$	695.4657	4.64	4.19E-04
PA 18:1/18:2 (1)	$[M - H]^-$	697.4813	4.75	>0.05 (SI)

PA 18:1/18:2 (2)	[M - H]-	697.4821	5.35	3.01E-02
PA 18:1/18:1 (1)	[M - H]-	699.4972	4.88	1.24E-05
PA 18:1/18:1 (2)	[M - H]-	699.4976	5.35	2.08E-04
PA 18:1/18:1 (3)	[M - H]-	699.4978	5.58	4.83E-05
PA 18:0/18:1	[M - H]-	701.5127	5.06	3.50E-04
PA 17:1/18:1	[M - H]-	685.4818	4.81	>0.05 (SI)
PA 17:0/18:1	[M - H]-	687.4975	4.95	1.96E-02
PA 34:3	[M - H]-	669.4501	4.63	3.13E-06
PA 16:0/18:2 (1)	[M - H]-	671.4666	5.19	>0.05 (SI)
PA 16:0/18:2 (2)	[M - H]-	671.4659	4.74	1.62E-03
PA 16:0/18:2 (3)	[M - H]-	671.4666	5.36	>0.05 (SI)
PA 16:0/18:1 (1)	[M - H]-	673.4813	4.86	2.73E-03
PA 16:0/18:1 (2)	[M - H]-	673.4816	5.35	1.18E-02
PA 16:0/16:1	[M - H]-	645.4502	4.72	>0.05 (SI)
PA 16:0/16:0	[M - H]-	647.4660	4.85	8.81E-04
MGMG 18:3 (1)	[M + HCOO]-	559.3124	3.70	5.68E-05
MGMG 18:3 (2)	[M + HCOO]-	559.3125	3.55	6.33E-12
MGMG 18:2	[M + HCOO]-	561.3282	3.94	1.47E-07
MGMG 18:1	[M + HCOO]-	563.3438	4.13	>0.05 (SI)
MGMG 18:0	[M + HCOO]-	565.3597	4.28	7.30E-06
MGMG 16:0	[M + HCOO]-	537.3282	4.11	6.98E-06
MGDG 18:3/18:3	[M + HCOO]-	819.5266	4.53	5.82E-08

MGDG 18:2/18:3	$[M + HCOO]^-$	821.5432	4.62	>0.05 (SI)
MGDG 18:2/18:2	$[M + HCOO]^-$	823.5580	4.72	3.75E-09
MGDG 18:1/18:2	$[M + HCOO]^-$	825.5735	4.83	2.21E-08
MGDG 18:1/18:1	$[M + HCOO]^-$	827.5890	4.97	2.75E-03
MGDG 18:0/18:1	$[M + HCOO]^-$	829.6046	5.12	>0.05 (SI)
MGDG 16:0/18:3	$[M + HCOO]^-$	797.5420	4.72	4.01E-03
MGDG 16:0/18:2	$[M + HCOO]^-$	799.5579	4.83	5.09E-03
MGDG 16:0/18:1	$[M + HCOO]^-$	801.5735	4.96	>0.05 (SI)
LPI 18:1 (1)	$[M - H]^-$	597.3051	3.18	4.46E-03
LPI 18:1 (2)	$[M - H]^-$	597.3049	3.48	6.83E-04
LPI 18:0	$[M - H]^-$	599.3204	3.98	2.22E-02
LPI 16:0	$[M - H]^-$	571.2892	3.05	7.22E-05
LPG 18:1	$[M - H]^-$	509.2884	3.76	4.27E-12
LPG 16:0	$[M - H]^-$	483.2726	3.48	3.68E-02
LPE 18:2 (1)	$[M - H]^-$	476.2780	3.63	1.05E-02
LPE 18:2 (2)	$[M - H]^-$	476.2781	3.42	4.56E-08
LPE 16:0	$[M - H]^-$	452.2779	3.93	>0.05 (SI)
LPA 18:2 (1)	$[M - H]^-$	433.2358	3.58	>0.05 (SI)
LPA 18:2 (2)	$[M - H]^-$	433.2357	3.40	>0.05 (SI)
LPA 18:1 (1)	$[M - H]^-$	435.2514	4.35	>0.05 (SI)
LPA 18:1 (2)	$[M - H]^-$	435.2513	3.97	3.42E-02
LPA 16:0	$[M - H]^-$	409.2358	3.91	2.17E-04

Cer 18:1;O3/26:0(2OH)	$[M + HCOO]^-$	754.6572	5.73	7.85E-05
Cer 18:0;O3/26:0(2OH)	$[M + HCOO]^-$	756.6730	5.87	1.82E-07
Cer 18:1;O3/25:0(2OH)	$[M - H]^-$	694.6360	5.59	4.47E-04
Cer 18:0;O3/25:0(2OH)	$[M + HCOO]^-$	742.6574	5.72	1.81E-07
Cer 18:1;O3/24:0(2OH)	$[M - H]^-$	680.6205	5.46	6.53E-03
Cer 18:1;O3/24:0(2OH)	$[M + HCOO]^-$	726.6260	5.46	5.86E-03
Cer 18:1;O3/24:0	$[M + HCOO]^-$	710.6311	5.61	1.87E-02
Cer 18:0;O3/24:0(2OH)	$[M - H]^-$	682.6362	5.59	1.26E-04
Cer 18:0;O3/24:0(2OH)	$[M + HCOO]^-$	728.6417	5.59	5.44E-05
Cer 18:0;O3/24:0	$[M + HCOO]^-$	712.6469	5.75	>0.05 (SI)
Cer 18:1;O3/23:0(2OH)	$[M + HCOO]^-$	712.6103	5.35	1.04E-02
Cer 18:1;O3/23:0(2OH)	$[M - H]^-$	666.6048	5.35	2.26E-02
Cer 18:0;O3/23:0(2OH)	$[M + HCOO]^-$	714.6263	5.46	4.14E-06
Cer 18:1;O3/22:0(2OH)	$[M + HCOO]^-$	698.5944	5.24	>0.05 (SI)
Cer 18:1;O3/22:0(2OH)	$[M - H]^-$	652.5891	5.24	>0.05 (SI)
Cer 18:1;O3/22:0	$[M + HCOO]^-$	682.5992	5.37	3.20E-02
Cer 18:0;O3/22:0(2OH)	$[M - H]^-$	654.6046	5.35	1.30E-02
Cer 18:0;O3/22:0(2OH)	$[M + HCOO]^-$	700.6099	5.35	8.71E-03
Cer 18:0;O3/22:0	$[M + HCOO]^-$	684.6152	5.49	>0.05 (SI)
Cer 18:1;O3/20:0(2OH)	$[M + HCOO]^-$	670.5632	5.04	5.35E-04
Cer 18:0;O3/16:0	$[M + HCOO]^-$	600.5200	4.89	>0.05 (SI)
ASG 29:2;O;Hex;FA 18:2 (1)	$[M + HCOO]^-$	881.6517	5.52	1.33E-02

ASG 29:2;O;Hex;FA 18:2 (2)	[M + HCOO]$^-$	881.6517	5.62	1.51E-07
ASG 29:2;O;Hex;FA 18:1	[M + HCOO]$^-$	883.6670	5.85	>0.05 (SI)
ASG 28:1;O;Hex;FA 18:2	[M + HCOO]$^-$	869.6516	5.63	2.15E-02
ASG 27:1;O;Hex;FA 18:1 (1)	[M + HCOO]$^-$	857.6521	5.69	4.39E-05
ASG 27:1;O;Hex;FA 18:1 (2)	[M + HCOO]$^-$	857.6517	5.83	1.65E-04

SI – Statistically insignificant; TG – triacylglycerol; ST – Sterols; PI – Phosphatidylinositol; PC – Phosphatidylcholine; NAE – N-Acylethanolamine; LPC – Lysophosphatidylcholine; LPE – Lysophosphatidylethanolamine; HexCer – Hexosylceramide; DGDG – Digalactosyldiacylglycerol; DG O- – Ether-linked Diacylglycerol; DG – Diacylglycerol; ASG – Acylated Sterol Glycoside; SQDG – Sulfoquinovosyldiacylglycerol; FA – Fatty Acid; PG – Phosphatidylglycerol; PE – Phosphatidylethanolamine; PA – Phosphatidic Acid; MGMG – Monogalactosylmonoacylglycerol; MGDG – Monogalactosyldiacylglycerol; LPI – Lysophosphatidylinositol; LPG – Lysophosphatidylglycerol; LPA – Lysophosphatidic Acid; Cer – Ceramide.

Table S5. Annotated lipids in cacao pod husk with ionization mode-specific ANOVA results.

**Name**	**Adduct**	***m/z***	**Rt(min)**	***p*-value (ANOVA)**
TG 18:2/18:2/18:3	[M + NH_4]$^+$	894.7526	6.58	>0.05 (SI)
TG 18:2/18:2/18:2	[M + NH_4]$^+$	896.7676	6.91	2.90E-05
TG 16:0/18:2/18:3	[M + NH_4]$^+$	870.7520	6.91	>0.05 (SI)
TG 16:0/18:2/18:2	[M + NH_4]$^+$	872.7675	7.24	6.89E-04
TG 18:1/18:2/18:2	[M + NH_4]$^+$	898.7828	7.26	9.02E-11
TG 17:0/18:2/18:2	[M + NH_4]$^+$	886.7830	7.43	>0.05 (SI)
TG 16:0/16:0/18:2	[M + NH_4]$^+$	848.7673	7.56	>0.05 (SI)
TG 16:0/18:1/18:2	[M + NH_4]$^+$	874.7828	7.58	3.94E-08
TG 18:1/18:1/18:2	[M + NH_4]$^+$	900.7987	7.61	3.36E-11
TG 16:0/16:0/18:1	[M + NH_4]$^+$	850.7827	7.85	>0.05 (SI)

TG 18:1/18:1/18:1	$[M + NH_4]^+$	902.8139	7.89	1.93E-03
TG 16:0/18:0/18:1	$[M + NH_4]^+$	878.8140	8.09	2.46E-02
TG 20:0/18:1/18:2	$[M + NH_4]^+$	930.8454	8.11	>0.05 (SI)
TG 17:0/18:0/18:1	$[M + NH_4]^+$	892.8297	8.19	6.63E-03
TG 18:1/20:0/18:1	$[M + NH_4]^+$	932.8605	8.28	>0.05 (SI)
TG 18:0/18:0/18:0	$[M + NH_4]^+$	908.8611	8.43	>0.05 (SI)
TG 18:0/20:0/18:1	$[M + NH_4]^+$	934.8765	8.43	>0.05 (SI)
TG 18:0/22:0/18:1	$[M + NH_4]^+$	962.9086	8.58	2.51E-02
ST 29:1;O (1)	$[M + H - H_2O]^+$	397.3823	6.33	4.24E-03
ST 29:1;O (2)	$[M + H - H_2O]^+$	397.3824	6.11	5.34E-05
ST 29:1;O (3)	$[M + H - H_2O]^+$	397.3824	5.96	>0.05 (SI)
ST 29:1;O (4)	$[M + H - H_2O]^+$	397.3828	7.23	3.80E-03
ST 29:1;O (5)	$[M + H - H_2O]^+$	397.3824	5.73	2.37E-04
ST 29:1;O (6)	$[M + H - H_2O]^+$	397.3824	6.97	2.12E-03
ST 29:1;O (7)	$[M + H - H_2O]^+$	397.3825	8.12	8.43E-04
ST 29:1;O (8)	$[M + H - H_2O]^+$	397.3825	5.63	3.16E-07
ST 29:1;O (9)	$[M + H - H_2O]^+$	397.3826	6.67	1.31E-04
ST 29:1;O (10)	$[M + H - H_2O]^+$	397.3827	8.34	4.05E-13
ST 29:1;O (11)	$[M + H - H_2O]^+$	397.3827	5.30	7.91E-03
SE 29:1/18:3 (1)	$[M + NH_4]^+$	692.6328	7.89	>0.05 (SI)
SE 29:1/18:3 (2)	$[M + NH_4]^+$	692.6328	6.33	6.75E-03
SE 29:1/18:2	$[M + NH_4]^+$	694.6486	8.12	6.87E-04

SE 29:1/18:1	$[M + NH_4]^+$	696.6647	8.34	1.78E-16
SE 28:1/18:2	$[M + NH_4]^+$	680.6332	8.03	3.67E-03
PC 38:5	$[M + H]^+$	808.5829	4.78	1.51E-04
PC 38:4	$[M + H]^+$	810.5985	4.90	5.60E-08
PC 38:3	$[M + H]^+$	812.6148	5.11	2.11E-02
PC 38:2	$[M + H]^+$	814.6300	5.39	>0.05 (SI)
PC 37:3	$[M + H]^+$	798.5989	5.12	2.09E-02
PC 37:2 (1)	$[M + H]^+$	800.6143	5.19	2.28E-04
PC 37:2 (2)	$[M + H]^+$	800.6149	5.28	>0.05 (SI)
PC 36:6	$[M + H]^+$	778.5364	4.54	1.89E-03
PC 36:5	$[M + H]^+$	780.5513	4.65	>0.05 (SI)
	$[M + HCOO]^-$	824.5446	4.67	7.05E-04
PC 36:4	$[M + H]^+$	782.5665	4.77	>0.05 (SI)
	$[M + HCOO]^-$	826.5602	4.77	>0.05 (SI)
PC 36:3 (1)	$[M + H]^+$	784.5823	4.89	1.29E-05
PC 36:3 (2)	$[M + H]^+$	784.5829	5.10	2.04E-06
PC 36:3 (3)	$[M + H]^+$	784.5833	5.19	3.16E-05
PC 36:2 (1)	$[M + H]^+$	786.5989	5.10	>0.05 (SI)
	$[M + HCOO]^-$	830.5915	5.09	1.22E-02
PC 36:2 (2)	$[M + H]^+$	786.5987	5.19	>0.05 (SI)
PC 36:2 (3)	$[M + H]^+$	786.5979	5.05	2.27E-02
PC 36:1	$[M + H]^+$	788.6146	5.26	2.98E-06

PC 35:4	$[M + H]^{+}$	768.5524	4.64	>0.05 (SI)
PC 35:3	$[M + H]^{+}$	770.5675	4.84	>0.05 (SI)
	$[M + HCOO]^{-}$	814.5605	4.85	1.15E-03
PC 35:2 (1)	$[M + H]^{+}$	772.5834	4.99	>0.05 (SI)
	$[M + HCOO]^{-}$	816.5763	5.00	>0.05 (SI)
PC 35:2 (2)	$[M + H]^{+}$	772.5829	5.09	>0.05 (SI)
PC 35:1	$[M + H]^{+}$	774.5987	5.19	2.86E-05
PC 34:3 (1)	$[M + H]^{+}$	756.5516	4.77	2.28E-04
	$[M + HCOO]^{-}$	800.5446	4.79	2.07E-05
PC 34:3 (2)	$[M + H]^{+}$	756.5523	4.99	>0.05 (SI)
PC 34:2 (1)	$[M + H]^{+}$	758.5676	5.19	>0.05 (SI)
PC 34:2 (2)	$[M + H]^{+}$	758.5670	4.89	>0.05 (SI)
PC 34:1 (1)	$[M + H]^{+}$	760.5832	5.19	2.33E-11
PC 34:1 (2)	$[M + H]^{+}$	760.5826	5.04	8.26E-10
PC 33:3	$[M + H]^{+}$	742.5369	4.78	2.98E-03
PC 33:2 (1)	$[M + H]^{+}$	744.5524	4.91	>0.05 (SI)
PC 33:2 (2)	$[M + H]^{+}$	744.5523	4.81	>0.05 (SI)
	$[M + HCOO]^{-}$	788.5450	4.83	>0.05 (SI)
PC 33:1	$[M + H]^{+}$	746.5685	4.96	>0.05 (SI)
PC 32:2 (1)	$[M + H]^{+}$	730.5367	4.84	>0.05 (SI)
PC 32:2 (2)	$[M + H]^{+}$	730.5367	4.74	>0.05 (SI)
PC 32:1	$[M + H]^{+}$	732.5525	4.86	1.97E-02

PC 32:0	$[M + H]^+$	734.5685	5.04	7.41E-03
PC 31:1	$[M + H]^+$	718.5372	4.89	1.18E-10
	$[M + HCOO]^-$	776.5449	4.87	4.90E-03
NAE 9:0	$[M + H]^+$	202.1799	0.92	>0.05 (SI)
NAE 20:2	$[M + H]^+$	352.3207	4.16	2.65E-04
NAE 18:2	$[M + H]^+$	324.2894	4.16	>0.05 (SI)
LPC 18:2 (1)	$[M + H]^+$	520.3392	3.73	>0.05 (SI)
	[M + HCOO]-	564.3311	3.74	>0.05 (SI)
LPC 18:2 (2)	$[M + H]^+$	520.3394	3.56	>0.05 (SI)
LPC 18:1	$[M + H]^+$	522.3549	4.01	2.05E-05
	$[M + HCOO]^-$	566.3465	4.00	>0.05 (SI)
LPC 18:0	$[M + H]^+$	524.3704	4.22	1.08E-08
LPC 16:0	$[M + H]^+$	496.3392	3.97	1.18E-03
HexCer 18:2;2O/20:1	$[M + H]^+$	752.6017	4.90	5.68E-03
HexCer 18:2;2O/18:1	$[M + H]^+$	724.5708	4.75	1.29E-02
HexCer 18:2;2O/16:1	$[M + H]^+$	696.5397	4.63	>0.05 (SI)
DGDG 18:3/18:3	$[M + NH_4]^+$	954.6124	4.42	>0.05 (SI)
	$[M + HCOO]^-$	981.5786	4.42	>0.05 (SI)
DGDG 18:1/18:3	$[M + NH_4]^+$	958.6437	4.58	1.04E-04
DGDG 36:3	$[M + NH_4]^+$	960.6596	4.68	8.90E-03
	$[M + HCOO]^-$	987.6259	4.68	>0.05 (SI)
DGDG 18:0/18:2	$[M + NH_4]^+$	962.6754	4.78	2.60E-07

	$[M + HCOO]^-$	989.6411	4.79	2.04E-06
DGDG 16:0/18:3	$[M + NH_4]^+$	932.6276	4.57	>0.05 (SI)
	$[M + HCOO]^-$	959.5947	4.57	>0.05 (SI)
DGDG 16:0/18:2	$[M + NH_4]^+$	934.6432	4.66	5.18E-06
	$[M + HCOO]^-$	961.6099	4.66	8.30E-05
DGDG 16:0/18:1	$[M + NH_4]^+$	936.6592	4.76	3.78E-07
	$[M + HCOO]^-$	963.6256	4.76	1.22E-06
DG 18:1/18:1	$[M + NH_4]^+$	638.5710	5.38	2.80E-03
DG 18:0/18:0	$[M + NH_4]^+$	642.6025	5.84	>0.05 (SI)
DG 16:0/18:1	$[M + NH_4]^+$	612.5554	5.35	1.17E-03
DG 16:0/18:0	$[M + NH_4]^+$	614.5713	5.57	4.66E-06
DG 16:0/16:0	$[M + NH_4]^+$	586.5402	5.33	>0.05 (SI)
DG 24:0/18:2	$[M + NH_4]^+$	722.6644	6.26	>0.05 (SI)
DG 23:0/18:2	$[M + NH_4]^+$	708.6490	6.09	>0.05 (SI)
DG 22:0/18:2	$[M + NH_4]^+$	694.6332	5.93	>0.05 (SI)
DG 18:2/18:3	$[M + NH_4]^+$	632.5239	4.90	1.01E-01
DG 18:1/18:2	$[M + NH_4]^+$	636.5554	5.19	7.97E-09
ASG 29:1;O;Hex;FA 18:2	$[M + NH_4]^+$	856.6998	5.73	2.15E-09
ASG 29:1;O;Hex;FA 18:1	$[M + NH_4]^+$	858.7157	5.96	8.97E-09
	$[M + HCOO]^-$	885.6826	5.96	2.01E-09
ASG 29:1;O;Hex;FA 18:0	$[M + NH_4]^+$	860.7310	6.27	6.59E-06
ASG 28:1;O;Hex;FA 16:0	$[M + NH_4]^+$	818.6852	5.85	>0.05 (SI)

	[M + HCOO]$^-$	845.6514	5.85	>0.05 (SI)
SQDG 36:3	[M - H]$^-$	843.5294	4.46	>0.05 (SI)
SQDG 36:6	[M - H]$^-$	837.4825	4.27	>0.05 (SI)
SQDG 18:2/18:3	[M - H]$^-$	839.4984	4.32	>0.05 (SI)
SQDG 36:4	[M - H]$^-$	841.5140	4.38	>0.05 (SI)
SQDG 18:0/18:2	[M - H]$^-$	845.5449	4.53	>0.05 (SI)
SQDG 17:0/18:3	[M - H]$^-$	829.5138	4.43	2.47E-03
SQDG 34:3	[M - H]$^-$	815.4985	4.38	>0.05 (SI)
SQDG 16:0/18:2	[M - H]$^-$	817.5142	4.44	1.38E-03
SQDG 16:0/16:1	[M - H]$^-$	791.4984	4.44	1.83E-06
SQDG 16:0/16:0	[M - H]$^-$	793.5141	4.50	1.74E-03
SQDG 16:0/15:1	[M - H]$^-$	777.4826	4.42	2.26E-09
SQDG 15:0/16:0	[M - H]$^-$	779.4987	4.46	9.72E-04
SQDG 17:0/16:1	[M - H]$^-$	805.5140	4.48	2.96E-05
PI 18:2/18:3	[M - H]$^-$	855.5025	4.36	1.61E-06
PI 18:2/18:2	[M - H]$^-$	857.5181	4.44	>0.05 (SI)
PI 18:1/18:2	[M - H]$^-$	859.5338	4.51	2.03E-14
PI 18:0/18:2	[M - H]$^-$	861.5493	4.61	1.19E-04
PI 18:0/18:1	[M - H]$^-$	863.5655	4.70	7.62E-06
PI 17:0/18:2	[M - H]$^-$	847.5339	4.55	6.26E-04
PI 16:0/18:3	[M - H]$^-$	831.5028	4.44	6.20E-10
PI 16:0/18:3;O	[M - H]$^-$	847.4985	4.19	>0.05 (SI)

PI 16:0/18:2	$[M - H]^-$	833.5183	4.50	>0.05 (SI)
PI 16:0/18:2;O	$[M - H]^-$	849.5139	4.23	>0.05 (SI)
PI 16:0/18:1	$[M - H]^-$	835.5338	4.59	1.44E-12
PI 16:0/16:1	$[M - H]^-$	807.5027	4.48	2.53E-10
PG 18:2/18:2	$[M - H]^-$	769.5026	4.47	>0.05 (SI)
PG 18:1/18:2	$[M - H]^-$	771.5181	4.56	8.00E-04
PG 16:0/18:3	$[M - H]^-$	743.4869	4.46	3.72E-03
PG 16:0/18:2	$[M - H]^-$	745.5027	4.55	>0.05 (SI)
PG 16:0/18:1	$[M - H]^-$	747.5184	4.63	1.88E-02
PG 16:0/18:0	$[M - H]^-$	749.5340	4.75	1.63E-03
PG 16:0/17:0	$[M - H]^-$	735.5187	4.69	>0.05 (SI)
PG 16:0/16:1	$[M - H]^-$	719.4865	4.53	>0.05 (SI)
PG 16:0/16:0	$[M - H]^-$	721.5025	4.62	>0.05 (SI)
PG 16:0/15:1	$[M - H]^-$	705.4717	4.51	1.07E-08
PG 15:0/16:0	$[M - H]^-$	707.4875	4.57	4.57E-04
PG 15:2/16:0	$[M - H]^-$	703.4562	4.31	>0.05 (SI)
PG 16:0/18:3;O	$[M - H]^-$	759.4822	4.21	3.53E-04
PE 24:0/22:3;O	$[M - H]^-$	896.6752	5.64	7.59E-07
PE 28:0/18:2	$[M - H]^-$	882.6962	6.10	>0.05 (SI)
PE 27:0/18:2	$[M - H]^-$	868.6799	5.92	>0.05 (SI)
PE 26:0/18:2	$[M - H]^-$	854.6641	5.78	>0.05 (SI)
PE 25:0/18:3	$[M - H]^-$	838.6334	5.45	5.85E-03

PE 25:0/18:2	[M - H]-	840.6483	5.63	>0.05 (SI)
PE 24:0/18:2	[M - H]-	826.6331	5.51	>0.05 (SI)
PE 24:0/18:3	[M - H]-	824.6176	5.34	>0.05 (SI)
PE 23:0/18:3	[M - H]-	810.6025	5.23	1.33E-02
PE 23:0/18:2	[M - H]-	812.6174	5.39	>0.05 (SI)
PE 18:2/22:3;O	[M - H]-	808.5498	4.75	1.66E-08
PE 22:0/18:2	[M - H]-	798.6024	5.28	>0.05 (SI)
PE 21:0/18:2	[M - H]-	784.5864	5.19	>0.05 (SI)
PE 16:0/22:3;O	[M - H]-	784.5500	4.86	2.03E-08
PE 20:0/18:2	[M - H]-	770.5708	5.09	>0.05 (SI)
PE 18:3/18:3	[M - H]-	734.4769	4.50	>0.05 (SI)
PE 18:2/18:3 (1)	[M - H]-	736.4925	4.59	>0.05 (SI)
PE 18:2/18:3 (2)	[M - H]-	736.4928	4.33	9.46E-06
PE 18:2/18:2 (1)	[M - H]-	738.5083	4.40	4.13E-04
PE 18:2/18:2 (2)	[M - H]-	738.5079	4.68	>0.05 (SI)
PE 18:1/18:2 (1)	[M - H]-	740.5237	4.46	2.34E-10
PE 18:1/18:2 (2)	[M - H]-	740.5237	4.78	4.36E-03
PE 18:0/18:2	[M - H]-	742.5394	4.92	>0.05 (SI)
PE 18:0/18:1	[M - H]-	744.5551	5.06	1.15E-06
PE 17:1/18:2	[M - H]-	726.5081	4.74	>0.05 (SI)
PE 17:0/18:2	[M - H]-	728.5239	4.84	>0.05 (SI)
PE 16:0/18:3	[M - H]-	712.4926	4.67	5.24E-03

PE 16:0/18:2	$[M - H]^-$	714.5079	4.77	>0.05 (SI)
PE 16:0/18:1	$[M - H]^-$	716.5236	4.89	3.18E-09
PE 16:0/16:1	$[M - H]^-$	688.4924	4.75	>0.05 (SI)
PE 15:0/18:2	$[M - H]^-$	700.4922	4.71	>0.05 (SI)
PA 18:2/18:3 (1)	$[M - H]^-$	693.4503	4.71	1.92E-07
PA 18:2/18:3 (2)	$[M - H]^-$	693.4501	4.81	2.83E-03
PA 18:2/18:2 (1)	$[M - H]^-$	695.4662	4.81	>0.05 (SI)
PA 18:2/18:2 (2)	$[M - H]^-$	695.4660	4.73	2.60E-03
PA 18:1/18:2 (1)	$[M - H]^-$	697.4819	4.85	>0.05 (SI)
PA 18:1/18:2 (2)	$[M - H]^-$	697.4816	4.94	1.54E-04
PA 18:0/18:2	$[M - H]^-$	699.4976	5.05	>0.05 (SI)
PA 17:0/18:2	$[M - H]^-$	685.4819	4.95	1.17E-04
PA 16:0/18:3 (1)	$[M - H]^-$	669.4504	4.73	1.21E-08
PA 16:0/18:3 (2)	$[M - H]^-$	669.4503	4.81	2.01E-08
PA 16:0/18:2 (1)	$[M - H]^-$	671.4664	4.45	>0.05 (SI)
PA 16:0/18:2 (2)	$[M - H]^-$	671.4658	4.94	>0.05 (SI)
PA 16:0/18:2 (3)	$[M - H]^-$	671.4664	5.88	2.01E-02
PA 16:0/18:2 (4)	$[M - H]^-$	671.4661	4.85	3.36E-04
PA 16:0/18:1	$[M - H]^-$	673.4819	5.03	9.67E-04
PA 15:0/18:2	$[M - H]^-$	657.4508	4.79	9.78E-03
PA 16:0/16:1	$[M - H]^-$	645.4506	4.82	3.50E-06
MGMG 18:3 (1)	$[M + HCOO]^-$	559.3127	3.74	>0.05 (SI)

MGMG 18:3 (2)	$[M + HCOO]^-$	559.3127	3.62	>0.05 (SI)
MGDG 18:3/18:3	$[M + HCOO]^-$	819.5262	4.54	>0.05 (SI)
MGDG 18:2/18:3	$[M + HCOO]^-$	821.5453	4.61	>0.05 (SI)
MGDG 36:4	$[M + HCOO]^-$	823.5578	4.73	>0.05 (SI)
MGDG 18:1/18:2	$[M + HCOO]^-$	825.5731	4.85	3.99E-03
MGDG 16:0/18:3	$[M + HCOO]^-$	797.5422	4.73	>0.05 (SI)
MGDG 16:0/18:2	$[M + HCOO]^-$	799.5577	4.83	1.87E-02
LPI 16:0	$[M - H]^-$	571.2893	3.18	6.01E-03
LPE 20:0	$[M - H]^-$	508.3408	4.35	2.89E-05
LPE 18:2 (1)	$[M - H]^-$	476.2781	3.53	>0.05 (SI)
LPE 18:2 (2)	$[M - H]^-$	476.2783	3.71	>0.05 (SI)
LPE 18:1	$[M - H]^-$	478.2942	4.03	>0.05 (SI)
LPE 18:0	$[M - H]^-$	480.3096	4.20	6.65E-05
LPE 16:0	$[M - H]^-$	452.2781	3.97	2.03E-03
LPA 18:2	$[M - H]^-$	433.2358	3.81	>0.05 (SI)
FA 26:0(2OH)	$[M - H]^-$	411.3841	5.03	7.94E-06
FA 25:0(2OH)	$[M - H]^-$	397.3684	4.95	1.63E-02
FA 24:0(2OH)	$[M - H]^-$	383.3527	4.86	1.69E-03
FA 22:0(2OH)	$[M - H]^-$	355.3214	4.70	7.29E-05
DGDG 17:0/18:2	$[M + HCOO]^-$	975.6260	4.72	1.94E-05
Cer 18:1;O3/26:0(2OH)	$[M + HCOO]^-$	754.6572	5.73	>0.05 (SI)
Cer 18:0;O3/26:0(2OH)	$[M + HCOO]^-$	756.6734	5.88	>0.05 (SI)

	$[M - H]^-$	710.6675	5.87	>0.05 (SI)
Cer 18:0;O3/25:0(2OH)	$[M + HCOO]^-$	742.6572	5.73	>0.05 (SI)
	$[M - H]^-$	696.6518	5.72	>0.05 (SI)
Cer 18:0;O3/25:0	$[M + HCOO]^-$	726.6625	5.91	>0.05 (SI)
Cer 18:1;O3/24:0(2OH)	$[M - H]^-$	680.6204	5.47	>0.05 (SI)
	$[M + HCOO]^-$	726.6260	5.47	>0.05 (SI)
Cer 18:0;O3/24:0(2OH)	$[M - H]^-$	682.6359	5.59	>0.05 (SI)
	$[M + HCOO]^-$	728.6414	5.59	>0.05 (SI)
Cer 18:0;O3/24:0	$[M + HCOO]^-$	712.6465	5.76	>0.05 (SI)
Cer 18:0;O3/23:0(2OH)	$[M + HCOO]^-$	714.6260	5.47	>0.05 (SI)
	$[M - H]^-$	668.6206	5.46	>0.05 (SI)
Cer 18:1;O3/22:0(2OH)	$[M + HCOO]^-$	698.5945	5.25	>0.05 (SI)
Cer 18:0;O3/22:0(2OH)	$[M + HCOO]^-$	700.6101	5.36	>0.05 (SI)
Cer 18:1;3O/24:0	$[M + HCOO]^-$	710.6310	5.62	9.10E-04
ASG 28:1;O;Hex;FA 18:2	$[M + HCOO]^-$	869.6514	5.64	1.61E-05
ASG 27:1;O;Hex;FA 18:1	$[M + HCOO]^-$	857.6517	5.84	8.39E-04
ADGGA 16:0/16:0/18:2	$[M - H]^-$	1005.7607	5.60	>0.05 (SI)

SI – Statistically insignificant; TG – Triacylglycerol; ST – Sterol; SE – Steryl Ester; PC – Phosphatidylcholine; NAE – N-Acylethanolamine; LPC – Lysophosphatidylcholine; LPE – Lysophosphatidylethanolamine; LPI – Lysophosphatidylinositol; LPA – Lysophosphatidic Acid; PI – Phosphatidylinositol; PG – Phosphatidylglycerol; PE – Phosphatidylethanolamine; PA – Phosphatidic Acid; DG – Diacylglycerol; SQDG – Sulfoquinovosyldiacylglycerol; MGDG – Monogalactosyldiacylglycerol; MGMG – Monogalactosylmonoacylglycerol; DGDG – Digalactosyldiacylglycerol; HexCer – Hexosylceramide; Cer – Ceramide; ASG – Acylated Sterol Glycoside; FA – Fatty Acid; ADGGA – Acyl-Diacylglyceryl Glucuronide.

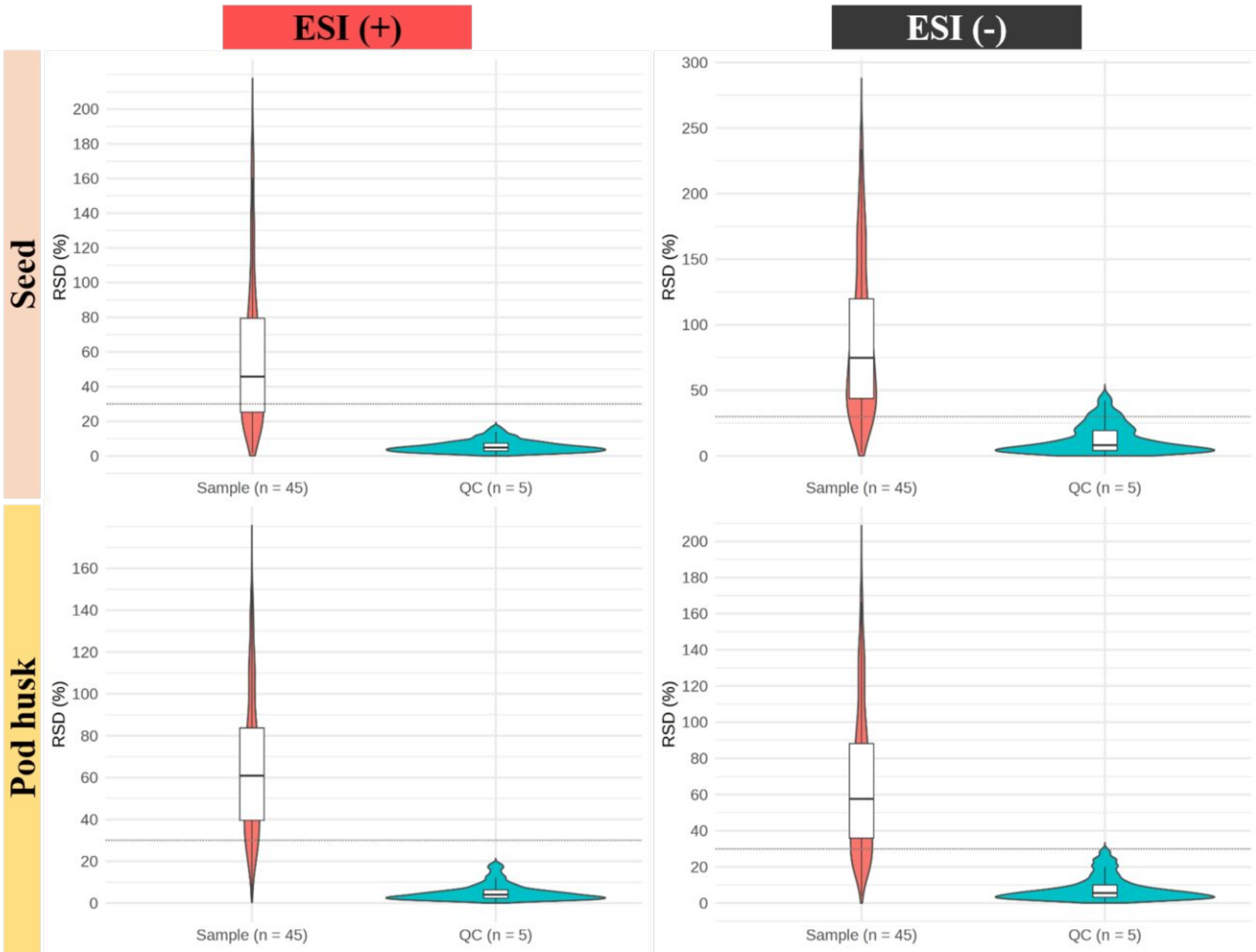

Figure S1. RSD comparison between QC and biological samples in each analytical batch.

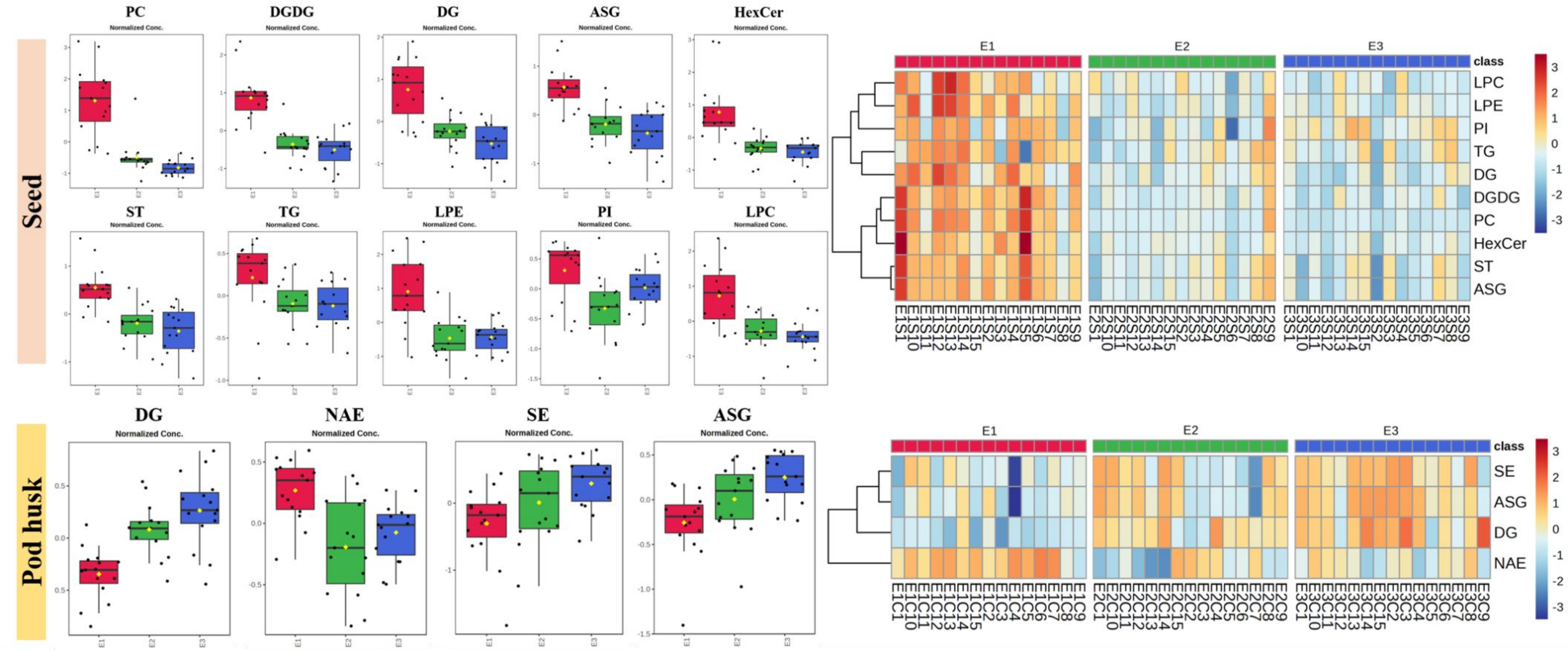

Figure S2. Boxplots and a heatmap of significant classes for cacao seed and CPH in ESI (+). Boxplot colors (red – E1; green – E2; blue – E3). Sample name codes: E – maturation stage; S – seeds; C – CPH. Heatmap parameters: normalized data; autoscale features; euclidean; ward clustering; 'Do not cluster'.

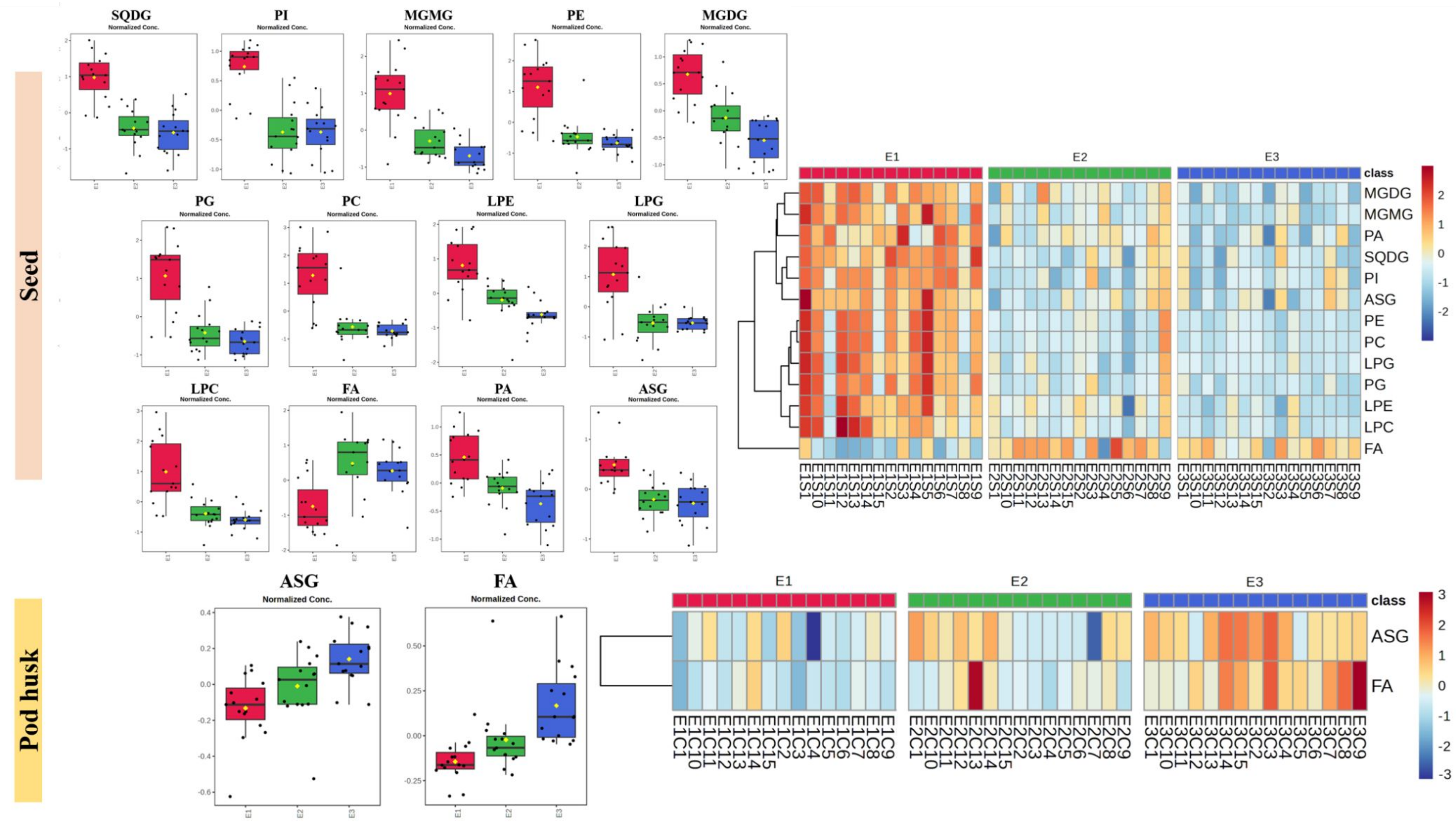

Figure S3. Boxplots and a heatmap of significant classes for cacao seed and CPH in ESI (-). Boxplot colors (red – E1; green – E2; blue – E3). Sample name codes: E – maturation stage; S – seeds; C – CPH. Heatmap parameters: normalized data; autoscale features; euclidean; ward clustering; 'Do not cluster'.

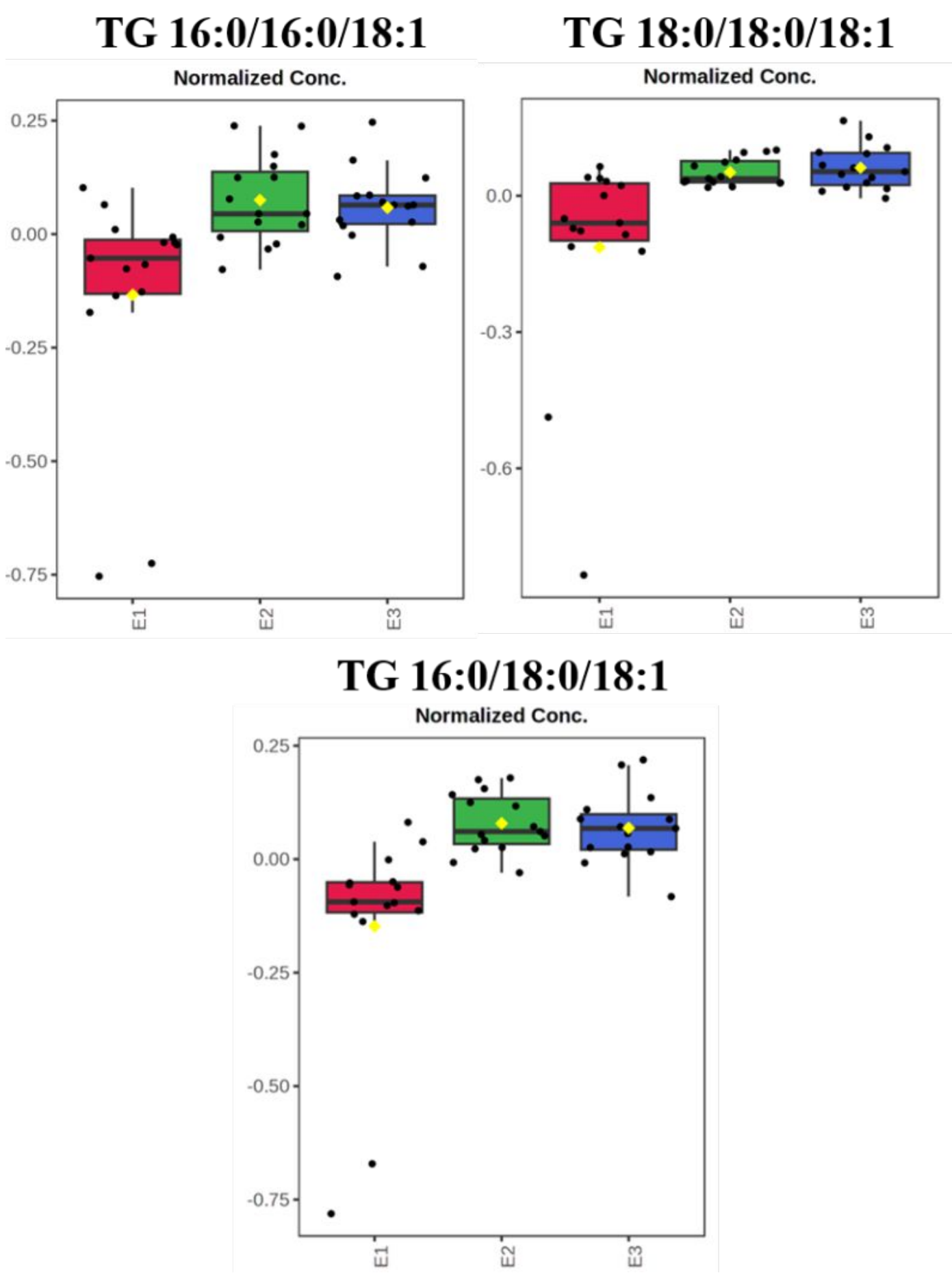

Figure S4. Boxplots of the main TGs of cocoa butter in ESI (+) of cacao seeds.

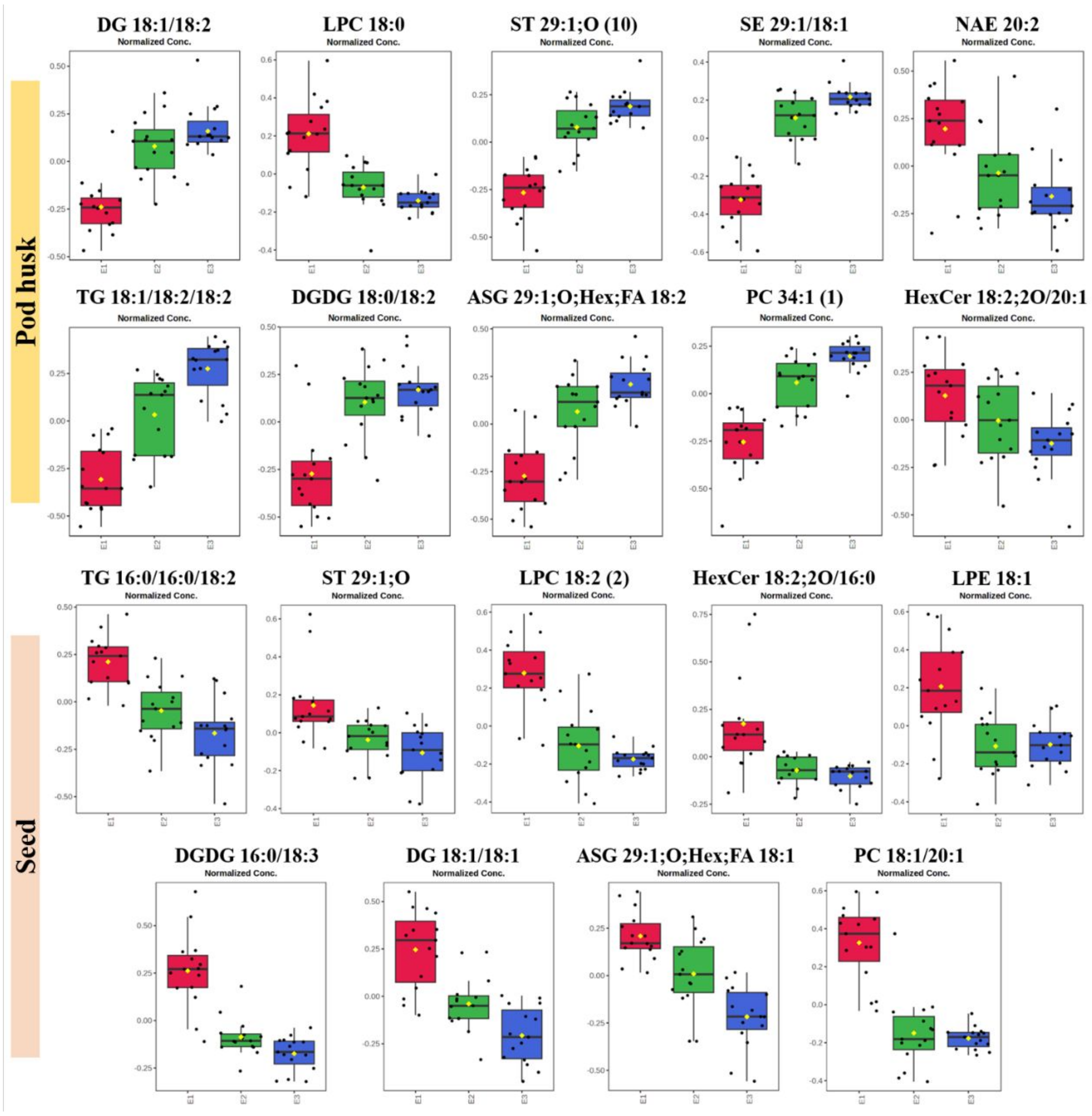

Figure S5. Boxplot of the main annotated feature of each class in ESI (+) of CPH and cacao seeds. Boxplot colors (red – E1; green – E2; blue – E3).

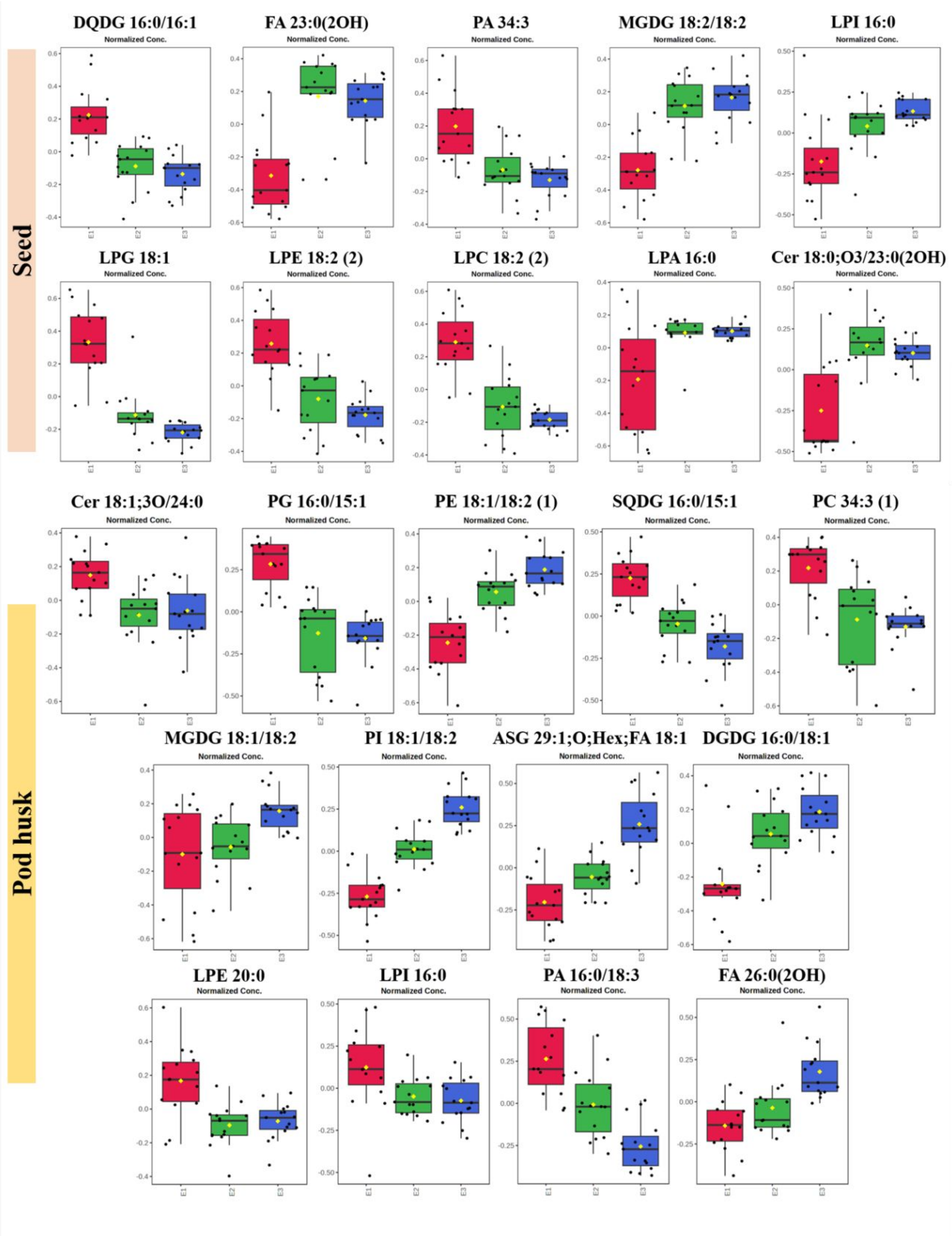

Figure S6. Boxplot of the main annotated feature of each class in ESI (-) of CPH and cacao seeds. Boxplot colors (red – E1; green – E2; blue – E3).
